# Artificial light-harvesting n-type porphyrin for panchromatic organic photovoltaic devices†
†Electronic supplementary information (ESI) available: ^1^H and ^13^C NMR, MALDI-TOF-MS, TGA and PL spectra, SCLC graph and photovoltaic properties are provided in detail. See DOI: 10.1039/c7sc01275f



**DOI:** 10.1039/c7sc01275f

**Published:** 2017-05-16

**Authors:** Wisnu Tantyo Hadmojo, Dajeong Yim, Havid Aqoma, Du Yeol Ryu, Tae Joo Shin, Hyun Woo Kim, Eojin Hwang, Woo-Dong Jang, In Hwan Jung, Sung-Yeon Jang

**Affiliations:** a Department of Chemistry , Kookmin University , 77 Jeongneung-ro, Seongbuk-gu , Seoul 02707 , Republic of Korea . Email: ihjung@kookmin.ac.kr ; Email: syjang@kookmin.ac.kr; b Department of Chemistry , Yonsei University , 50 Yonsei-ro, Seodaemun-gu , Seoul , Republic of Korea . Email: wdjang@yonsei.ac.kr; c UNIST Central Research Facilities , School of Natural Science , Ulsan National Institute of Science and Technology (UNIST) , 50 UNIST-gil, Eonyang-eup, Ulju-gun , Ulsan , Republic of Korea; d Center for Molecular Modeling and Simulation , Korea Research Institute of Chemical Technology (KRICT) , 141 Gajeong-ro, Yuseong-gu , Daejeon , Republic of Korea

## Abstract

A near-infrared-harvesting n-type porphyrin-based acceptor for organic photovoltaics (OPVs) was developed. The n-type acceptor, PDI–P_Zn_–PDI, was designed by connecting a zinc porphyrin (P_Zn_) core to two perylenediimide (PDI) wings through ethyne bridges. A narrow bandgap of 1.27 eV was achieved through the extended π-conjugation and intramolecular charge transfer between the strongly electron-donating P_Zn_ core and the electron-accepting PDI wings. A bulk heterojunction (BHJ) structured photovoltaic device fabricated from PDI–P_Zn_–PDI with PTB7-Th exhibited panchromatic photon-to-current conversion from 350 to 900 nm. A power conversion efficiency of 5.25% with a remarkably low *E*_loss_ of 0.54 eV was achieved by optimizing the nanomorphology of the BHJ films by adding pyridine and by controlling the ZnO/BHJ interfacial properties.

## Abstract 2

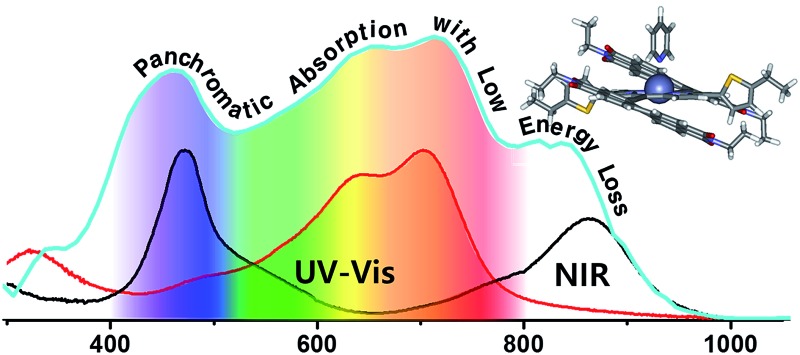
We developed a novel NIR-harvesting n-type porphyrin derivative, PDI–P_Zn_–PDI, that shows a low bandgap of 1.27 eV. Panchromatic absorption was extended to the NIR area with a significantly low energy loss of 0.54 eV which led to promising photovoltaic performance.

## Introduction

Porphyrin derivatives are essential pigments for natural light-harvesting systems.^1^ Owing to their high chemical stability and versatile redox activity, porphyrins have become a central chemical species in many fundamental biological functions.^2^ In particular, light-harvesting antenna complexes (LHCs) in plants and bacteria are composed of several well-organized porphyrin derivatives.^1,3–5^ The porphyrin derivatives in the LHCs efficiently absorb visible photons and energy is collected in the reaction center through the excitation energy transfer relay.^6^ Charge separation occurs at the reaction center through the cooperation of a pair of porphyrin derivatives. These critical roles for porphyrin in light-to-charge conversion in biological systems have inspired artificial light-harvesting systems such as dye-sensitized solar cells (DSSCs)^6^ and organic photovoltaic (OPV) devices.^7^

The high optical absorbability of porphyrin derivatives has afforded them respectable performances as photoactive materials for solar cells. A record-high power conversion efficiency (PCE) for DSSCs (13%) was achieved using porphyrin-based dyes.^8–10^ Recently, porphyrin derivatives have also achieved significant performances as the photoactive materials in OPVs.^11–14^ However, successful reports of using porphyrin derivatives in solar cells have mainly focused on the design of p-type donor materials.^8–13^ Although Li *et al.* recently reported a highly promising n-type porphyrin acceptor,^14^ the relatively few reports on the applications of n-type porphyrin-based acceptors for solar cells have not demonstrated meaningful solar cell performances (PCE <1% in DSSCs and OPVs).^15,16^ Therefore, the development of high-performance n-type porphyrin-based photoactive materials is a challenging and unexplored area in artificial light-harvesting system research. Considering the adequate electron-transport properties of porphyrins in natural systems, the performance of porphyrin-based acceptors can be improved by judicious molecular design.

**Scheme 1:**
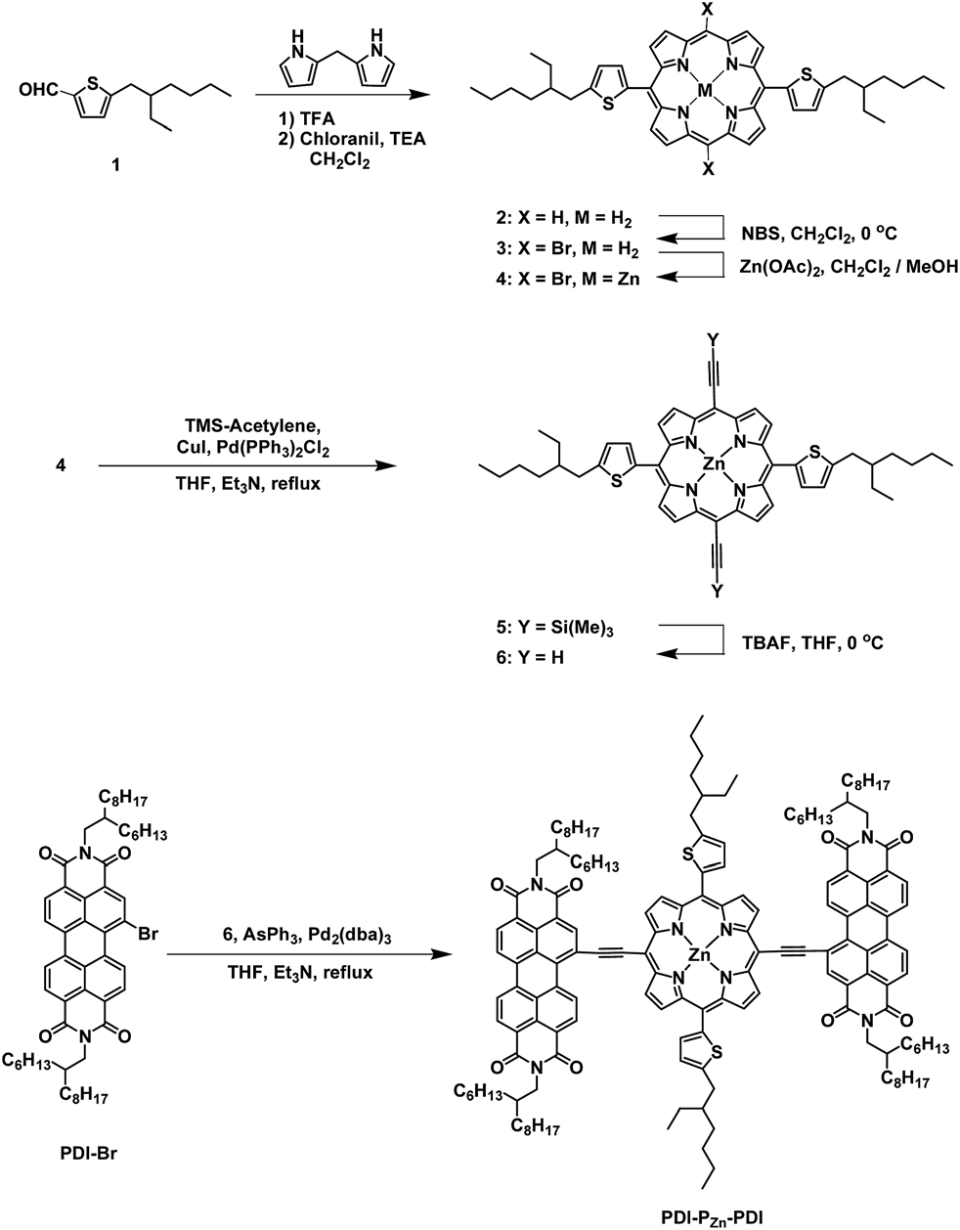
Synthesis of PDI–P_Zn_–PDI.

In this study, we synthesized a novel n-type porphyrin derivative (PDI–P_Zn_–PDI; Scheme 1) that exhibits near infrared (NIR) absorption. The narrow bandgap of 1.27 eV and good electron-transport properties were achieved as a result of the strong intramolecular charge transfer (ICT) between the P_Zn_ cores and PDI wings. Using PDI–P_Zn_–PDI as an acceptor, panchromatic OPV devices with a remarkably low energy loss (*E*_loss_) of 0.54 eV were achieved.

## Results and discussion

### Synthesis

The synthetic procedure for PDI–P_Zn_–PDI is outlined in Scheme 1. The porphyrin core was synthesized using modified procedures from the literature.^17–20^ A thiophene-bearing porphyrin skeleton (**2**) was prepared by an acid-catalyzed cyclization reaction between dipyrromethane and 5-(2-ethylhexyl)thiophene-2-carbaldehyde (**1**) and successive oxidation. Bromine was introduced to the meso-positions of **2** by using *N*-bromosuccinimide (NBS) to afford **3**. Trimethylsilyl acetylene was introduced *via* the Sonogashira coupling reaction to yield **5**. Finally, PDI–P_Zn_–PDI was prepared by the Sonogashira coupling of **6** and PDI–Br.

The PDI–P_Zn_–PDI acceptor was unambiguously characterized by ^1^H and ^13^C NMR and matrix-assisted laser desorption ionization time-of-flight mass spectrometry (MALDI-TOF-MS) (Fig. S1–S9†). Thermal gravimetric analysis (TGA) demonstrated the excellent thermal stability of PDI–P_Zn_–PDI, which exhibited a 5% weight loss temperature (*T*_5d_) of 427 °C (Fig. S10†) under a N_2_ atmosphere.

### Optical and electrochemical properties

**Fig. 1:**
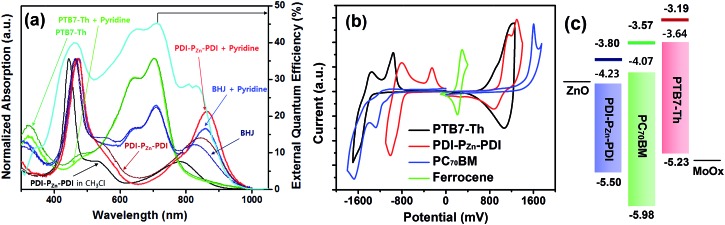
(a) UV-vis-NIR absorption spectra of PDI–P_Zn_–PDI, PTB7-Th and PTB7-Th:PDI–P_Zn_–PDI BHJ films (with or without pyridine). The cyan line indicates the EQE of the BHJ OPV devices. (b) CV results of PDI–P_Zn_–PDI and PTB7-Th. (c) Band diagrams of PDI–P_Zn_–PDI and PTB7-Th determined from electrochemical (lines) and optical (boxes) analyses.

Fig. 1a shows the absorption spectra for the chemical species used in this study. The optical absorption spectrum of PDI–P_Zn_–PDI exhibits two distinct, strong absorption peaks that stem from the Soret (400–600 nm) and Q-bands (700–1000 nm). The extended conjugation and intramolecular charge transfer (ICT) interactions between the strong electron-donating P_Zn_ core and the electron-accepting PDI wings pushed the Q-band absorption into the NIR region. The optical bandgap (*E*_g_) of PDI–P_Zn_–PDI was 1.27 eV (Table 1). In addition, the ethyne bridges enhanced the planarity of the molecule by releasing the steric hindrance between P_Zn_ and PDI. Notably, our PDI–P_Zn_–PDI is the first PDI-containing NIR-absorbing material. Although PDI is a popular moiety for designing n-type acceptors, its suboptimal π-electron delocalization due to its high resonance energy and twisted dihedral angle with adjacent molecules limits its potential as a narrow bandgap acceptor for OPVs.^21–23^ Unlike other PDI-containing acceptors, our PDI–P_Zn_–PDI effectively overcomes those limitations through extended π-conjugation due to its planar molecular geometry.

**Table 1:** Summary of optical and electrochemical properties

	*λ*_max_ (nm)		*E* opt g ^*c*^ (eV)	*E* CV g ^*d*^ (eV)	*E*_HOMO_ ^*e*^ (eV)	*E* CV LUMO ^*f*^ (eV)	*E* opt LUMO ^*g*^ (eV)
	Solution^*a*^	Film^*b*^					
PTB7-Th	—	699	1.59	2.04	–5.23	–3.19	–3.64
PC_70_BM	—	400, 504	1.91	2.41	–5.98	–3.57	–4.07
PDI–P_Zn_–PDI	445, 780	467, 850	1.27	1.70	–5.50	–3.80	–4.23

^*a*^Dilute chloroform solution.

^*b*^Film on a quartz plate, formed by spin-coating a 1 wt% chloroform solution for 30 s at 1500 rpm.

^*c*^Bandgap calculated from the film-state absorption onset wavelength.

^*d*^Bandgap between *E*_HOMO_ and *E*_LUMO_.

^*e*^HOMO levels determined from the *E*_onset_ of the first oxidation potential of ferrocene, –4.8 eV.

^*f*^LUMO levels from the *E*_onset_ of the first reduction potential.

^*g*^LUMO levels calculated from HOMO levels and *E*optg.

**Fig. 2:**
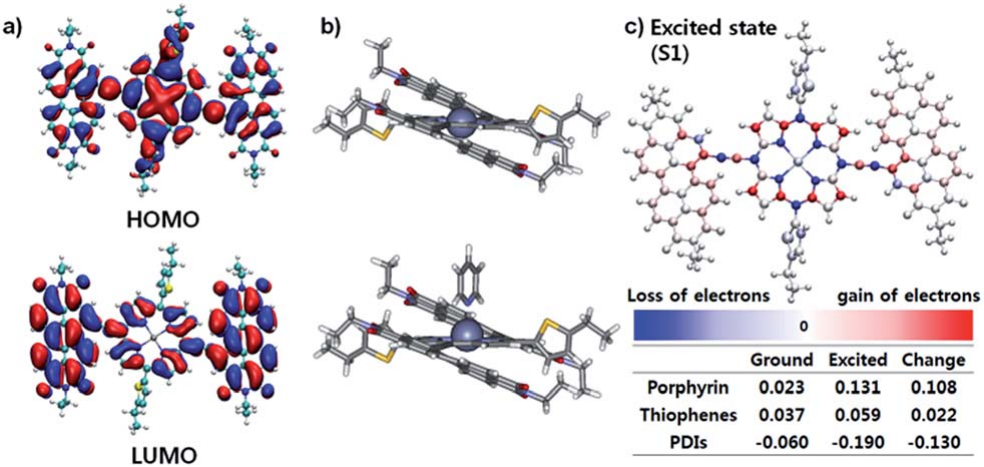
Geometric structures of PDI–P_Zn_–PDI calculated by the DFT method using B3LYP/6-31G(d, p). (a) HOMO and LUMO of PDI–P_Zn_–PDI, (b) side view of the tube shape, with and without pyridine, and (c) charge variation from the ground state (S_0_) to the excited state (S_1_).

The effective ICT between the P_Zn_ core and PDI wings was strongly supported by the Frontier molecular orbitals determined from density functional theory (DFT) calculations with the B3LYP function employing the 6-31G(d, p) basis set.^24^ As shown in Fig. 2a, the HOMO is crowded in P_Zn_, with partial distribution in the PDI wings. Alternatively, the LUMO is delocalized throughout the entire PDI–P_Zn_–PDI, indicating strong ICT. The charge variation in the excited state (S_1_), which was simulated by the CAM-B3LYP functional for time-dependent DFT calculations,^25^ confirmed the strong charge transfer from the electron-donating P_Zn_ core to the electron-accepting PDI wings (0.130 e) (Fig. 2c). The electron-pushing characteristics of the alkylthiophenes substituents also helped the charge transfer. As shown in the side view of PDI–P_Zn_–PDI (Fig. 2b), the dihedral angle between PDI and the P_Zn_ core was only 20° because of the planarization effects of the ethyne bridges. This is a substantially more suppressed dihedral angle than those of other reported small molecule acceptors containing PDI units (40–60°).^21–23^

### Photovoltaic properties

To evaluate the capability of PDI–P_Zn_–PDI as a photoactive acceptor for OPV devices, we fabricated bulk heterojunction (BHJ) structured devices using poly([2,6′-4,8-di(5-ethylhexylthienyl)benzo[1,2-*b*;3,3-*b*]dithiophene]{3-fluoro-2[(2-ethylhexyl)carbonyl]thieno[3,4-*b*]thiophenediyl}) (PTB7-Th)^26^ as a donor counterpart because PTB7-Th shows high hole mobility and complementary absorption to PDI–P_Zn_–PDI. By combining PDI–P_Zn_–PDI and PTB7-Th, panchromatic absorption from 350 nm to 900 nm was achieved in the active layers (blue lines in Fig. 1a). The band diagram of the active materials in Fig. 1c was determined using an electrochemical method (Fig. 1b) and an optical method (Fig. 1a). The energy levels of PDI–P_Zn_–PDI and PTB7-Th were appropriately located for charge separation (∼0.6 eV).

**Fig. 3:**
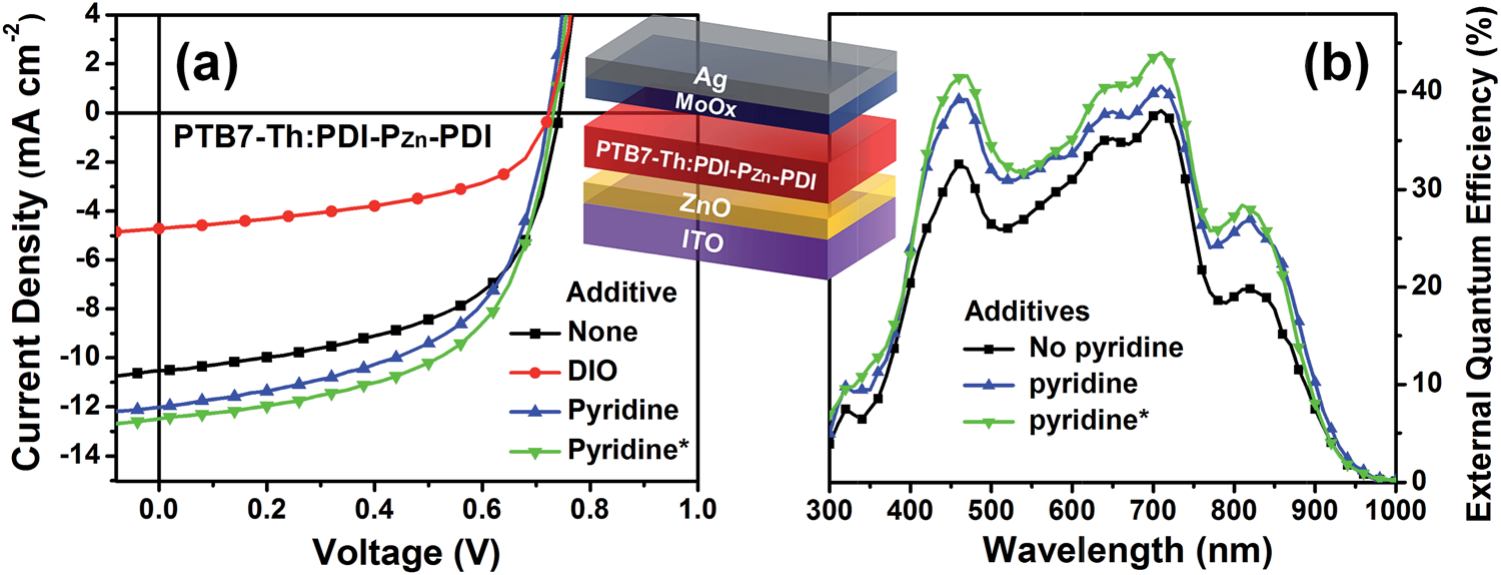
(a) The *J*–*V* characteristics of inverted-structure BHJ OPV devices (the inset shows the device architecture); (b) EQE of the PTB7-Th:PDI–P_Zn_–PDI photovoltaic devices corresponding to the *J*_SC_ values.

Inverted-structure devices (ITO/ZnO/PTB7-Th:PDI–P_Zn_–PDI/MoO_*x*_/Ag) were fabricated. The current density–voltage (*J*–*V*) characteristics of the inverted devices are shown in Fig. 3, S13 and S14,† and their photovoltaic parameters are summarized in Tables 2, S2 and S3.† The external quantum efficiency (EQE) spectra in Fig. 1a (cyan line) and 3b exhibit panchromatic photon-to-current conversion from 350 nm to 900 nm, revealing that the devices can efficiently collect charge from both PTB7-Th and PDI–P_Zn_–PDI. The effects of the donor/acceptor ratio were investigated, and a PTB7-Th : PDI–P_Zn_–PDI ratio of 1 : 1.4 showed optimum performance due to balanced optical absorption and charge collection (Fig. S14†). The interfacial charge transfer between the BHJ active layers and the ZnO electron-transport layers (ETLs) was optimized by chemical modification using 1,2-ethanedithiol (EDT), which is known to diminish surface defects on ZnO.^27,28^ This modification further improved the PCE to 5.25% with a *V*_OC_ of 0.73 V, a *J*_SC_ of 12.75 mA cm^–2^, and a FF of 0.56. There was no considerable performance difference to the conventional structure device (Fig. S16 and Table S4†).

**Table 2:** Summary of photovoltaic properties

Additive	*V*_OC_ ^*a*^	*J*_SC_ ^*b*^	FF	PCE	Mobility^*c*^		*μ*_h_/*μ*_e_
					*μ*_h_ (×10^–4^)	*μ*_e_ (×10^–4^)	
No	0.74	10.46	0.56	4.30%	23.4	2.25	10.4
DIO	0.72	4.72	0.51	1.74%			
Pyridine	0.72	12.08	0.55	4.81%	13.5	4.17	3.25
Pyridine^*d*^	0.73	12.76	0.56	5.25%			

^*a*^V.

^*b*^mA cm^–2^.

^*c*^cm^–2^ V^–1^ s^–1^.

^*d*^ZnO ETL was treated with EDT.

Notably, the *V*_OC_ value of 0.73 V was remarkably high considering the *E*_g_ of PDI–P_Zn_–PDI is only 1.27 eV. The *E*_loss_ value, defined as *E*_loss_ = *E*_g_ – *q*⋅*V*_OC_ (where *q* is the elementary charge),^29,30^ was 0.54 eV, which is much lower than other reported values.^31^

**Fig. 4:**
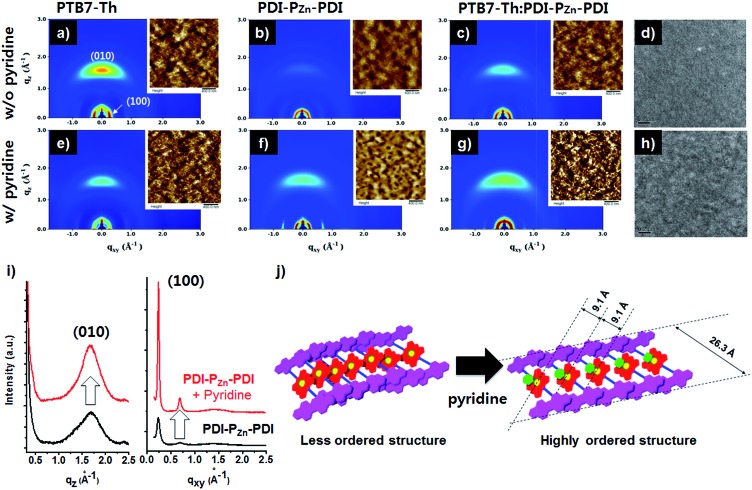
2D-GIXD images of pristine and blend films: (a) PTB7-Th, (b) PDI–P_Zn_–PDI and (c) PTB7-Th : PDI–P_Zn_–PDI (1.0 : 1.2 w/w) without pyridine, and (e) PTB7-Th, (f) PDI–P_Zn_–PDI and (g) PTB7-Th : PDI–P_Zn_–PDI (1.0 : 1.2 w/w) with 0.8 vol% pyridine. Inset images are the corresponding AFM images. TEM images of PTB7-Th : PDI–P_Zn_–PDI (1.0 : 1.2 w/w) (d) without or (h) with pyridine. (i) Out-of-plane and in-plane line-cut of the PDI–P_Zn_–PDI film with or without pyridine and (j) PDI–P_Zn_–PDI ordering with pyridine.

Incorporating a small amount of additive significantly influenced the device performance (Fig. 3). We applied two types of additive, 1,8-diiodooctane (DIO) and pyridine (Fig. S13†). DIO is known as the best-selling additive for BHJ layers^32–34^ and presumably improves miscibility between donors and acceptors. However, the addition of 0.5% DIO to the PTB7-Th:PDI–P_Zn_–PDI active layer significantly diminished the device performance. In contrast, adding 0.8 vol% of pyridine considerably improved the device performance (Table 2). The Lewis acidic zinc porphyrin is well known to possess a strong affinity to pyridine derivatives through axial coordination.^7,35,36^ We can expect that the axial coordination of pyridine to P_Zn_ provides dual effects, *i.e.*, electronic and steric effects. Firstly, the coordination of pyridine to P_Zn_ improves the electron-donating characteristics of the P_Zn_ core because of the lone-pair electrons in pyridine.^35^ Secondly, the coordination of pyridine covers one side of the porphyrin face, which can induce the rearrangement of the assembled structure (Fig. 4j).^37^ The absorption of the PDI–P_Zn_–PDI films (black and red lines) and BHJ films (blue and dark blue lines) was clearly altered by the addition of pyridine, as shown in Fig. 1a. The considerable red-shift in the spectra of those films (20–30 nm) upon the addition of pyridine supported such effects. The nanomorphology studies described in the following section further confirm the evolution in the molecular packing.

### Morphological studies

Fig. 4 exhibits the results from a two-dimensional grazing incidence X-ray diffraction (2D-GIXD) analysis of the BHJ active layers. Pristine PTB7-Th without pyridine displayed a strong face-on orientation along the out-of-plane (*Q*_*z*_) axis of 1.617 Å^–1^, corresponding to a π–π stacking distance of 3.89 Å (Fig. 4a). With the addition of pyridine, the ordering of PTB7-Th was reduced, while the *d*-spacing remained intact (3.89 Å, Fig. 4e). However, in the case of PDI–P_Zn_–PDI, the face-on stacking along the *Q*_*z*_ axis (010) was enhanced by the addition of pyridine, while the *d*-spacing slightly increased from 3.70 Å (*Q*_*z*_) to 3.75 Å (*Q*_*z*_). On the other hand, the in-plane (*Q*_*xy*_) axis ordering of PDI–P_Zn_–PDI was greatly enhanced by the addition of pyridine. As shown in Fig. 4i, the diffraction peaks at 0.24 and 0.68 Å^–1^ were greatly enhanced by the addition of pyridine. These results imply that pyridine promotes a new type of PDI–P_Zn_–PDI molecular ordering. The first diffraction peak (100) from the in-plane (*Q*_*xy*_) axis is consistent with the long-axis distance of PDI–P_Zn_–PDI (26.3 Å) (Fig. 4i and j), which reveals the molecular dimensions. Because the axial coordination of pyridine to P_Zn_ prevents the direct contact of the porphyrin cores, π–π stacking among P_Zn_ should be diminished. Instead, the π–π interaction among the PDI wings was predominant in the new molecular ordering. As mentioned above, the energy-minimized molecular structure obtained from DFT calculations indicated a 20° dihedral angle between PDI and P_Zn_. Considering the molecular structure of PDI–P_Zn_–PDI, the π–π stacking among PDI units is more favorable for the formation of long-range ordering. Based on the 2D-GIXD results, we propose plausible molecular stacking structures, as shown in Fig. 4j.

In PTB7-Th:PDI–P_Zn_–PDI blend films, intense face-on orientation with a diffraction peak along the *Q*_*z*_ axis (3.75 Å) was observed, revealing the formation of π–π interactions between PTB7-Th and PDI–P_Zn_–PDI in the face-on direction. On the other hand, diffraction patterns of both PTB7-Th and PDI–P_Zn_–PDI were retained in the in-plane (*Q*_*xy*_) axis. Moreover, the diffraction peaks in the in-plane (*Q*_*xy*_) axis direction of PDI–P_Zn_–PDI were strongly enhanced by the addition of pyridine, even in the BHJ films. These observations indicated that PDI–P_Zn_–PDI can form long-range ordering in BHJ films while maintaining strong π–π interactions with PTB7-Th.

Atomic force microscopy (AFM) images of pristine and BHJ films in the inset images of Fig. 4 support the nanomorphology suggested by the 2D-GIXD results. The root mean square roughness (*R*_RMS_) of the neat PDI–P_Zn_–PDI film significantly increased from 0.71 nm to 3.58 nm upon the addition of pyridine; however the *R*_RMS_ of PTB7-Th was nearly unchanged (0.97 nm *vs.* 1.02 nm). This indicates that pyridine considerably influences the interactions among PDI–P_Zn_–PDI molecules. In PTB7-Th:PDI–P_Zn_–PDI blend films, the film surface was more crumpled after the addition of pyridine, while the *R*_RMS_ value increased from 0.79 nm to 1.35 nm. The transmission electron microscope (TEM) images of the PTB7-Th:PDI–P_Zn_–PDI blend films (Fig. 4d and h) agree well with the results from the GIXD and AFM analyses. The addition of pyridine clearly led to the nanofibrillar structures of the PTB7-Th:PDI–P_Zn_–PDI blends (Fig. 4h). This well-developed nanoscale bicontinuous morphology facilitated charge separation/transport, thus enhancing the device performance.

The effects of nanomorphology on charge mobility were investigated by measuring the space-charge limited current (SCLC) (Fig. S17 and S18†), which strongly correlated with the charge mobility of the active blend films. As indicated in the GIXD results, the hole mobility (*μ*_h_) of the blend films was diminished upon the addition of pyridine, whereas the electron mobility (*μ*_e_) was enhanced. As a result, the charge balance (*μ*_h_/*μ*_e_) was improved from 10.4 to 3.25 by the addition of pyridine. This result can be attributed to the decreased crystallinity of p-type PTB7-Th and the increased crystallinity of n-type PDI–P_Zn_–PDI due to the addition of pyridine.

## Conclusions

We developed a novel NIR-harvesting narrow-bandgap n-type porphyrin derivative, PDI–P_Zn_–PDI, and applied it as an acceptor for OPV devices. The narrow bandgap and good electron transport properties were due to the strong ICT between the P_Zn_ cores and PDI wings through planar bridging using acetylene units. Forming BHJ layers with PTB7-Th, which exhibits complementary absorption, led to efficient panchromatic photon-to-current conversion from 350 nm to 950 nm. The PCE of 5.25% was achieved by optimizing the nanomorphology by adding pyridine and the interfacial properties by chemical modification. This result is unprecedentedly high among OPV devices using porphyrin-based acceptors. Notably, our PDI–P_Zn_–PDI-based devices also displayed a remarkably low *E*_loss_ of 0.54 eV. This new type of NIR absorbing acceptor without a severe loss of *V*_OC_ promises further performance improvements in panchromatic OPV devices by the selection of other visible absorbing donors.
