# Supplementary material for: Artificial light-harvesting n-type porphyrin for panchromatic organic photovoltaic devices
Source: Chem Sci. 2017 May 16;8(7):5095–100. doi: 10.1039/c7sc01275f (PMC5613227; doi:10.1039/c7sc01275f)
Supplement: Supplementary file 1 [file SC-008-C7SC01275F-s001.pdf]

Electronic Supplementary Material (ESI) for Chemical Science.
This journal is © The Royal Society of Chemistry 2017

Electronic Supplementary Information

Artificial Light-Harvesting n-type Porphyrin for Panchromatic Organic Photovoltaic Devices

Wisnu Tantyo Hadmojo,[a,§] *Dajeong Yim,*[b,§] *Havid Aqoma,*[a] *Du Yeol Ryu,*[b] *Tae Joo Shin,*[c] *Hyun Woo Kim,*[d] *Eojin Hwang,*[b] *Woo-Dong Jang,**[b] *In Hwan Jung**[a] *and Sung-Yeon Jang**[a]

[a] Department of Chemistry, Kookmin University, 77 Jeongneung-ro, Seongbuk-gu, Seoul 136-702, Republic of Korea. E-mail: ihjung@kookmin.ac.kr & syjang@kookmin.ac.kr

[b] Department of Chemistry, Yonsei University, 50 Yonsei-ro, Seodaemun-gu, Seoul, Republic of Korea. E-mail: wdjang@yonsei.ac.kr

[c] UNIST Central Research Facilities & School of Natural Science, Ulsan National Institute of Science and Technology (UNIST), 50 UNIST-gil, Eonyang-eup, Ulju-gun, Ulsan, Republic of Korea

[d] Center for Molecular Modeling and Simulation, Korea Research Institute of Chemical Technology (KRICT), 141 Gajeong-ro, Yuseong-gu, Daejeon, Republic of Korea

Materials and Measurements

All commercially available reagents were reagent grade and used without further purification. Dichloromethane (CH_2Cl_2), tetrahydrofuran (THF), and *N,N*-Dimethylformamide (DMF) were freshly distilled before each use. ^1H and ^{13}C NMR spectra were recorded on a Bruker Advance DPX 400 spectrometer at 25 °C in $CDCl_3$ and THF-d_8. MALDI-TOF-MS was performed on a Bruker Daltonics LRF20 using dithranol (1,8,9-trihydroxyanthracene) and HCCA (α-cyano-4-hydroxycinnamic acid) as the matrix. Recycling SEC was performed on a JAI model LC9021 equipped with JAIGEL-1H, JAIGEL-2H and JAIGEL-3H columns using THF (DUKSAN) as the eluent. Cyclic voltammetry was performed on a BAS 100B/W electrochemical analyzer with a three-electrode cell in a 0.1 N Bu_4NBF_4 solution in acetonitrile at a scan rate of 50 mV/s. The polymer film was coated onto a Pt wire electrode by dipping the electrode into a polymer solution in chloroform. All measurements were calibrated against an internal standard of ferrocene (Fc), the ionization potential (IP) value of which is −4.8 eV for the Fc/Fc^+ redox system. The absorption spectra were measured on a SHIMADZU/UV-2550 model UV-visible spectrophotometer. Thermogravimetric analysis (TGA) was carried out on a Thermo plus EVO II TG 8120 instrument under N_2 atmosphere with a heating rate of 10 °C min^{-1}.

Computational details

All density functional theory (DFT) and time-dependent DFT (TDDFT) calculations were carried out with Q-Chem software package. We adopted B3LYP functional for DFT calculations while we chose CAM-B3LYP functional for TDDFT calculations to explain charge-transfer excitations. All atoms were described with 6-31G(d, p) basis set. After geometry optimizations at the ground state, frontier orbitals were visualized with VMD software. To quantify the amount of the charge transfer, the natural population analysis was performed for both ground and first excited state electron densities.

Two-dimensional grazing-incidence X-ray diffraction (2D-GIXD)

2D-GIXD measurements were performed under vacuum at the 6D beamline at Pohang Light Source, Korea. The samples were prepared on ZnO-modified Si substrates under the same conditions as those used for the fabrication of solar cell devices. The wavelength of the X-ray was 0.1651 nm and the incidence angle (i.e., the angle between the critical angle of the sample and that of Si) was 0.12°. The 2D-GIXRD patterns were recorded using a 2D-CCD detector (MX225-HS, Rayonix L.L.C., USA), and the X-ray irradiation time was set at 0.5 to 10 s, depending on the saturation level of the detector. The 2D-GIXRD images from the films were analyzed based on the relationship between the scattering vector (q) and the d spacing ($q = 2\pi/d$).

Device Fabrication

The inverted structure bulk heterojunction OPVs were fabricated with an architecture of ITO/ZnO/BHJ/MoOx/Ag. First, ZnO electron transporting layers (20 nm) were prepared by *in situ* ZnO sol-gel conversion on pre-cleaned ITO substrates following a reported procedure.[s1, s2] A precursor solution, a 0.45 M solution of zinc acetate dehydrate ($[Zn(CH_3COO)_2 \cdot 2H_2O]$, Sigma Aldrich, 99.9%, 5 g) and ethanolamine ($NH_2CH_2CH_2OH$, Sigma Aldrich, 99.5%, 1.35 mL) in 2-methoxyethanol ($CH_3OCH_2CH_2OH$, Sigma Aldrich, 99.8%, 50 mL), was spin-coated at 4000 rpm for 15 s in an ambient environment, followed by thermal treatment at a mild temperature (gradual increase from room temperature to 200 °C). For the chemical modification process, 1,2-ethanedithiol (EDT) solution (1 wt% in acetonitrile) was spin-coated on the substrate at 5000 rpm for 1 min. The bulk heterojunction active layers were achieved by spin-coating the PTB7-Th:PDI-P_{Zn}-PDI solutions with or without additives (DIO and pyridine). The donor was dissolved in chloroform and stirred for 1 hour in ambient environment. The donor density was 7.5 mg/mL. The thickness of the active layers was ~90

nm. Thin hole transporting layers, MoOx (8 nm), and a metal electrode, Ag (100 nm), were deposited by thermal evaporation at reduced pressure (10^{-6} bar). The conventional structure bulk heterojunction OPVs were fabricated with an architecture of ITO/PEDOT:PSS/BHJ/ZnO/Al. PEDOT:PSS, purchased from Heraeus (Clevios PH1000) was diluted in isopropanol (1:1 v/v) and spin-coated on ITO/glass substrates at 4000 rpm for 30s followed by annealing at 120°C for 20 min. The active layers were fabricated at same conditions with the inverted devices. ZnO nanoparticle was spin-coated on the active layers at 4000 rpm for 15 s. Finally, Al electrodes (100 nm) were deposited by thermal evaporation at reduced pressure (10^{-6} bar). The active area was measured to be 0.07 cm^2.

Device Analysis

The *J-V* characteristics of the devices were measured using a Keithley 2401 instrument and a solar simulator with a 150 W Xenon lamp (Newport) as the light source. The light intensity was set to AM 1.5G and calibrated using a monosilicon standard from the National Renewable Energy Laboratory (NREL). The incident photon to electron (IPCE) spectra were obtained by passing the output of a 400-W Xenon lamp through a monochromator using the appropriate order-sorting filters. The collimated output of the monochromator was measured through a 1-mm aperture with calibrated Newport 818-UV and 818-IR power meters (McScience, K3100 IQX). The IPCE values were collected at a chopping frequency of 1 Hz from 300 to 1100 nm. The charge mobilities of the active layers were measured by the steady-state space-charge-limited current (SCLC) method. The structures of hole-only and electron-only devices were ITO/PEDOT:PSS/active layer/MoOx/Ag and ITO/ZnO/active layer/ZnO/Al, respectively. The mobility of the blend films was determined by Mott-Gurney law in the SCLC trap free regime, by using the slope of $J^{1/2}$ vs V.

Synthesis

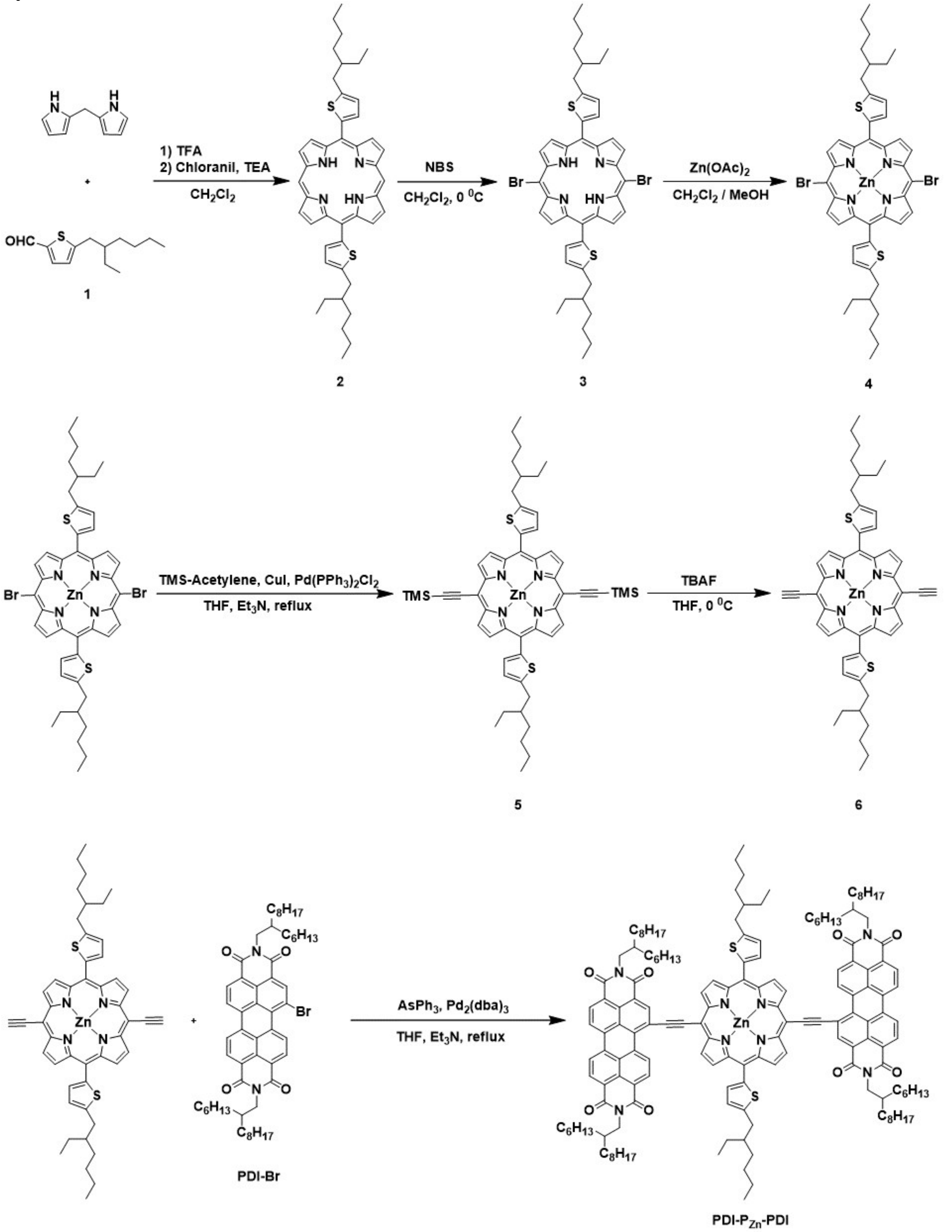

Scheme S1. Synthesis of PDI-P_{Zn}-PDI

1: To a solution of thiophene (10 mL, 0.125 mol) in dried THF (50 mL, cooled to -78 °C), *n*-BuLi (1.6 M in *n*-hexanes, 93.7 mL, 0.150 mol) was added dropwise and stirred for 1 h. After 2-ethylhexyl bromide (22.2 mL, 0.125 mol) was added dropwise to the mixture at -78 °C, the mixture was allowed to react at room temperature for several hours and then stirred at 80 °C overnight. The reaction was quenched with H_2O and extracted with ethyl acetate. The organic layer was collected and dried over $MgSO_4$. Then, 2-(2-ethylhexyl)thiophene (11.7 g, 48%) was obtained by vacuum distillation; the second fraction was collected and dissolved in dried THF (100 mL) in a 300 mL two-neck round-bottomed flask. The solution was cooled to -78 °C, and *n*-BuLi (1.6 M in *n*-hexanes, 41.2 mL, 65.9 mmol) was added dropwise to the reaction mixture. After the mixture was stirred at -78 °C for 1 h, DMF (5.6 mL, 71.9 mmol) was added, and the reaction solution was allowed to react at room temperature overnight. After the reaction was quenched with H_2O, the mixture was dissolved in ethyl acetate and washed with water (100 mL) three times. The organic layer was separated, and the solvent was evaporated under reduced pressure. The residue was purified using column chromatography with 2% ethyl acetate/hexanes, and the eluent was concentrated under vacuum to give **1** as a pale yellow oil (7.6 g, 57%): ^{1}H NMR (400 MHz, $CDCl_3$, 25 °C) δ= 9.81 (s, 1 H), 7.61-7.60 (d, 1 H, J = 4.0 Hz), 6.88-6.87 (d, 1 H, J = 4.0 Hz), 2.82-2.80 (d, 2 H, J = 8.0 Hz), 1.65-1.60 (m, 1 H), 1.37-1.28 (m, 8 H), 0.91-0.87 (m, 6 H). MALDI-TOF-MS: m/z: calcd. for $C_{13}H_{20}OS$: 224.12 $[M]^+$; found 222.78.

2: To a mixed solution of **1** (3 g, 13.4 mmol) and dipyrromethane (2 g, 13.7 mmol) in CH_2Cl_2 (900 mL), trifluoroacetic acid (TFA; 0.7 mL) was added and stirred for 2 h at 25 °C. Then, *p*-chloranil (8.2 g, 33.43 mmol) and triethylamine (TEA; 0.7 mL) were added and stirred overnight. The reaction mixture was concentrated and chromatographed in silica gel with 10% ethyl acetate/hexanes, followed by recrystallization from methanol to afford a purple powder (4.2 g, 45%): ^{1}H NMR (400 MHz, $CDCl_3$, 25 °C) δ= 10.28 (s, 2 H), 9.39-9.38 (d, 4 H, J = 4.0

Hz), 9.35-9.34 (d, 4 H, J = 4.0 Hz), 7.75-7.74 (d, 2 H, J = 4.0 Hz), 7.21-7.20 (d, 2 H, J = 4.0 Hz), 3.11-3.09 (d, 4 H, J = 8.0 Hz), 1.90-1.85 (m, 2 H), 1.65-1.59 (m, 8 H), 1.46-1.45 (m, 8 H), 1.10-1.06 (t, 6 H, J = 8.0 Hz), 1.02-0.98 (t, 6 H, J = 8.0 Hz), -2.96 (s, 2 H). MALDI-TOF-MS: m/z: calcd. for $C_{44}H_{50}N_4S_2$: 698.35 $[M]^+$; found 700.00.

3: *N*-Bromosuccinimide (NBS; 1.5 g, 8.45 mmol) was added to a solution of **2** (2.88 g, 4.12 mmol) in CH_2Cl_2 (200 mL) at 0 °C under N_2 and stirred for 15 min. The reaction mixture was quenched with acetone and chromatographed in silica gel with 30% CH_2Cl_2/hexanes to give **3**, a purple solid (2.5 g, 71%): ^1H NMR (400 MHz, $CDCl_3$, 25 °C) δ = 9.60-9.59 (d, 4 H, J = 4.0 Hz), 9.10-9.09 (d, 4 H, J = 4.0 Hz), 7.68-7.67 (d, 2 H, J = 4.0 Hz), 7.17-7.16 (d, 2 H, J = 4.0 Hz), 3.09-3.07 (d, 4 H, J = 8.0 Hz), 1.88-1.83 (m, 2 H), 1.62-1.58 (m, 8 H), 1.46-1.45 (m, 8 H), 1.09-1.05 (t, 6 H, J = 8.0 Hz), 1.02-0.98 (t, 6 H, J = 8.0 Hz), -2.64 (s, 2 H). MALDI-TOF-MS: m/z: calcd. for $C_{44}H_{48}Br_2N_4S_2$: 854.17 $[M]^+$; found 857.84.

4: Compound **3** (2.5 g, 2.92 mmol) was dissolved in CH_2Cl_2 (180 mL) in a 500 mL round-bottomed flask, and $Zn(OAc)_2$ (1.3 g, 5.84 mmol) in MeOH was added to the flask. After stirring at r.t., the mixture was extracted from CH_2Cl_2 and purified by column chromatography with CH_2Cl_2 as an eluent to give **4** as a blue-purple solid (2.4 g, 91%): ^1H NMR (400 MHz, $CDCl_3$, 25 °C) δ = 9.70-9.69 (d, 4 H, J = 4.0 Hz), 9.20-9.19 (d, 4 H, J = 4.0 Hz), 7.67-7.66 (d, 2 H, J = 4.0 Hz), 7.17-7.16 (d, 2 H, J = 4.0 Hz), 3.09-3.07 (d, 4 H, J = 8.0 Hz), 1.88-1.83 (m, 2 H), 1.64-1.57 (m, 8 H), 1.49-1.45 (m, 8 H), 1.10-1.06 (t, 6 H, J = 8.0 Hz), 1.02-0.99 (t, 6 H, J = 8.0 Hz). MALDI-TOF-MS: m/z: calcd. for $C_{44}H_{46}Br_2N_4S_2Zn$: 916.08 $[M]^+$; found 919.96.

5: **4** (2.4 g, 2.64 mmol), CuI (5 mg, 0.0264 mmol) and $Pd(PPh_3)_2Cl_2$ (18.5 mg, 0.0264 mmol) were mixed in a 300 mL two-neck round-bottomed flask. The flask was degassed under high vacuum and back-filled with N_2. TEA (60 mL) was added, and the reaction solution was stirred at 50 °C. After 1 h, trimethylsilylacetylene (1 mL, 6.60 mmol) was added to the flask through the septum, and the reaction was stirred at 70 °C overnight. The reaction mixture was washed

with water, and the organic layer was separated. After the solvent was evaporated under reduced pressure, the residue was purified using column chromatography with 25% CH_2Cl_2/hexanes to give **5** (1.8 g, 72%): ^{1}H NMR (400 MHz, $CDCl_3$, 25 °C) δ = 9.68-9.67 (d, 4 H, J = 4.0 Hz), 9.18-9.17 (d, 4 H, J = 4.0 Hz), 7.68-7.67 (d, 2 H, J = 4.0 Hz), 7.16-7.15 (d, 2 H, J = 4.0 Hz), 3.09-3.07 (d, 4 H, J = 8.0 Hz), 1.89-1.83 (m, 2 H), 1.64-1.45 (m, 16 H), 1.09-1.05 (t, 6 H, J = 8.0 Hz), 1.02-0.98 (t, 6 H, J = 8.0 Hz), 0.60 (s, 18 H). MALDI-TOF-MS: m/z: calcd. for $C_{54}H_{64}N_4S_2Si_2Zn$: 952.34 [M] $^+$; found 951.89.

6: Tetrabutylammonium fluoride (1 M in THF, 1.1 mL, 1.06 mmol) was added to a solution of **5** (461.8 mg, 0.48 mmol) in distilled THF (120 mL) in an ice bath. After the mixture was stirred at 0 °C for 30 min, the reaction was quenched with water and extracted with CH_2Cl_2. After the solvent was evaporated, the residue was purified by column chromatography with 50% CH_2Cl_2/hexanes to give **6** as a dark green powder: ^{1}H NMR (400 MHz, $CDCl_3$, 25 °C) δ = 9.73-9.72 (d, 4 H, J = 4.0 Hz), 9.22-9.21 (d, 4 H, J = 4.0 Hz), 7.69-7.68 (d, 2 H, J = 4.0 Hz), 7.17-7.16 (d, 2 H, J = 4.0 Hz), 4.18 (s, 2 H), 3.09-3.08 (d, 4 H, J = 4.0 Hz), 1.87-1.85 (m, 2 H), 1.64-1.46 (m, 16 H), 1.09-1.05 (t, 6 H, J = 8.0 Hz), 1.02-0.98 (t, 6 H, J = 8.0 Hz). MALDI-TOF-MS: m/z: calcd. for $C_{48}H_{48}N_4S_2Zn$: 808.26 [M] $^+$; found 810.93.

PDI-P$_{Zn}$-PDI: **6** (100 mg, 0.12 mmol), PDI-Br (250 mg, 0.27 mmol), $AsPh_3$ (3.8 mg, 0.012 mmol) and $Pd_2(dba)_3$ (11.3 mg, 0.012 mmol) were mixed in a 100 mL two-neck round-bottomed flask. The flask was degassed under high vacuum and back-filled with N_2 three times. TEA (5 mL) and THF (5 mL) were added to the flask, and the reaction was refluxed overnight. The reaction mixture was extracted with CH_2Cl_2, and the organic layer was evaporated *in vacuo*. The product was purified by silica gel column chromatography and recycling SEC with THF. After recrystallization, PDI-P$_{Zn}$-PDI was obtained as a dark green solid: ^{1}H NMR (400 MHz, THF-d_8, 25 °C) δ = 10.67-10.65 (d, 2 H, J = 8.0 Hz), 9.10-9.08 (m, 6 H), 8.99 (s, 6 H), 8.55 (s, 6 H), 8.38-8.36 (d, 2 H, J = 8.0 Hz), 8.08 (br, 2 H), 7.56-7.55 (d, 2 H, J = 4.0 Hz), 4.34-

4.33 (d, 4 H, J = 4.0 Hz), 4.25-4.24 (d, 4 H, J = 4.0 Hz), 3.34-3.33 (d, 4 H, J = 4.0 Hz), 2.20 (m, 2 H), 2.12 (m, 4 H), 1.60-1.25 (m, 118 H), 1.15-1.11 (t, 6 H, J = 8.0 Hz), 0.92-0.90 (m, 12 H), 0.83-0.82 (m, 12 H). ^{13}C NMR (100 MHz, $CDCl_3$, 25 °C) δ = 163.14, 163.04, 162.72, 162.33, 152.01, 151.24, 148.55, 139.77, 138.13, 134.46, 134.29, 134.14, 134.04, 133.54, 132.00, 131.04, 130.71, 130.40, 130.16, 128.43, 127.63, 126.91, 126.04, 125.42, 123.43, 122.58, 122.19, 121.56, 120.36, 117.37, 106.22, 100.85, 99.74, 44.28, 41.83, 36.66, 34.86, 33.19, 32.05, 31.63, 30.24, 29.92, 29.76, 29.52, 29.41, 26.47, 26.24, 23.53, 22.82, 14.62, 14.25, 11.32, 0.21. MALDI-TOF-MS: m/z: calcd. for $C_{160}H_{192}N_8O_8S_2Zn$: 2481.36 [M] +; found 2483.67.

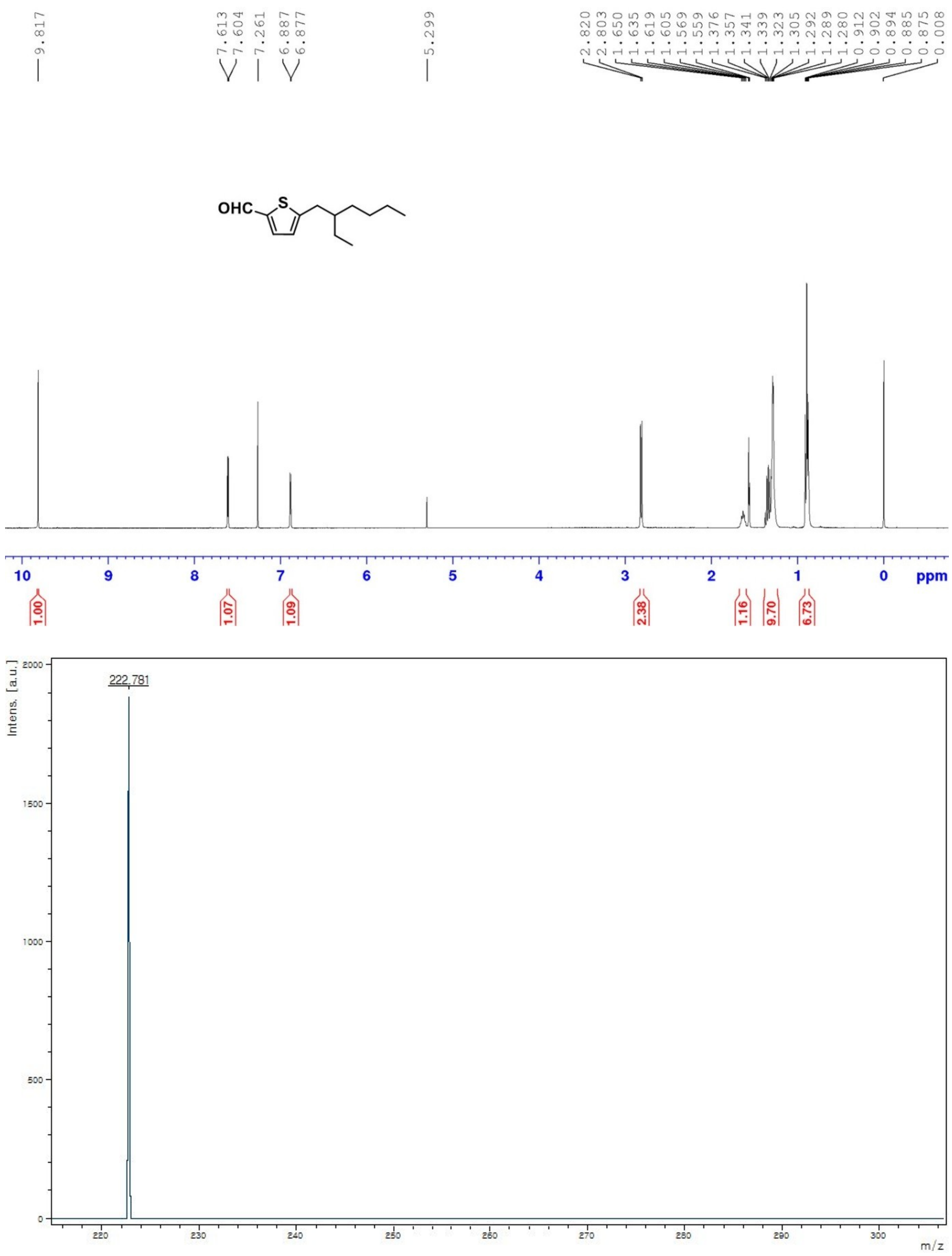

Figure S1. ^{1}H NMR (400 MHz, $CDCl_3$, 298 K) spectrum and MALDI-TOF-MS spectrum of compound **1**.

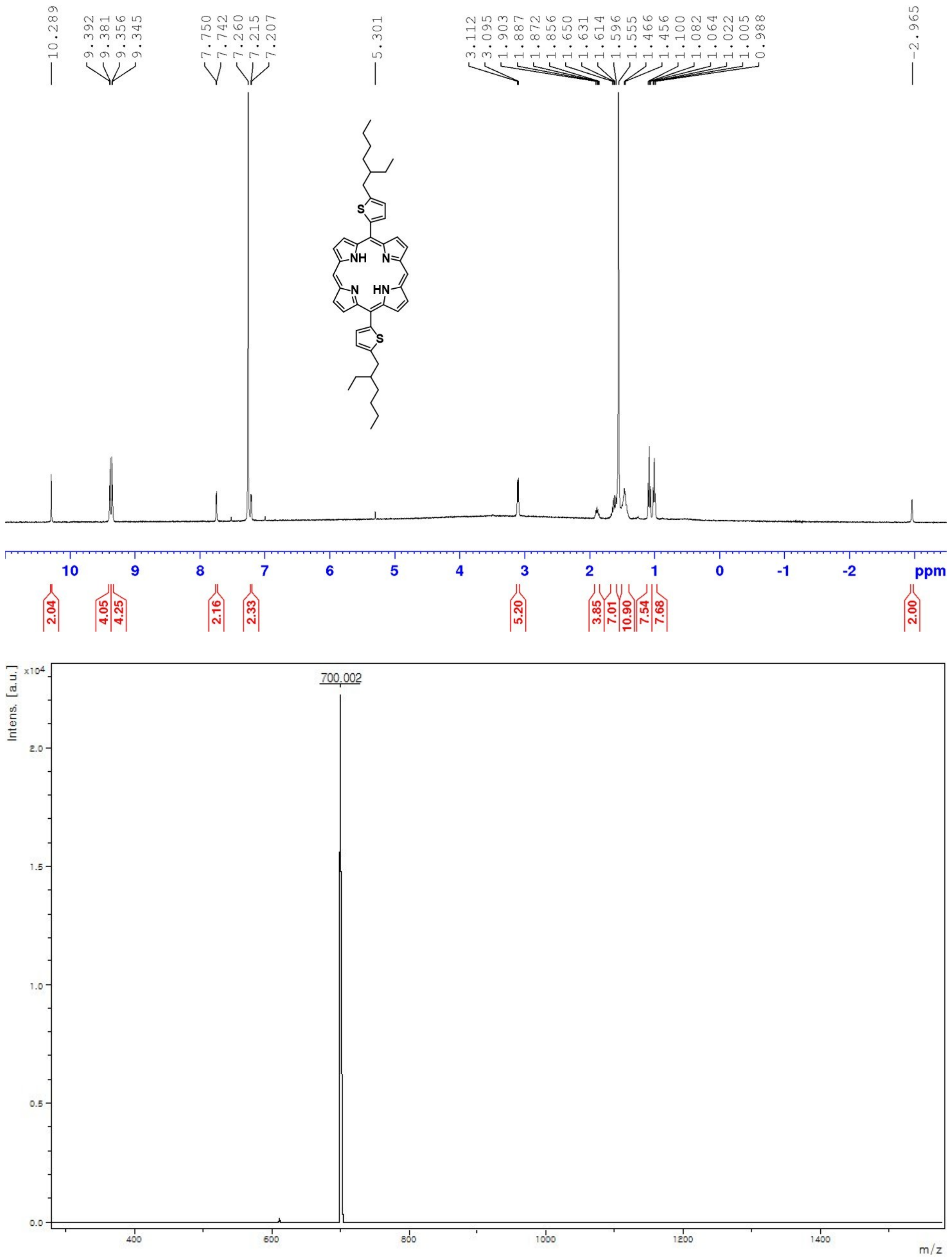

Figure S2. ^{1}H NMR (400 MHz, $CDCl_3$, 298 K) spectrum and MALDI-TOF-MS spectrum of compound **2**.

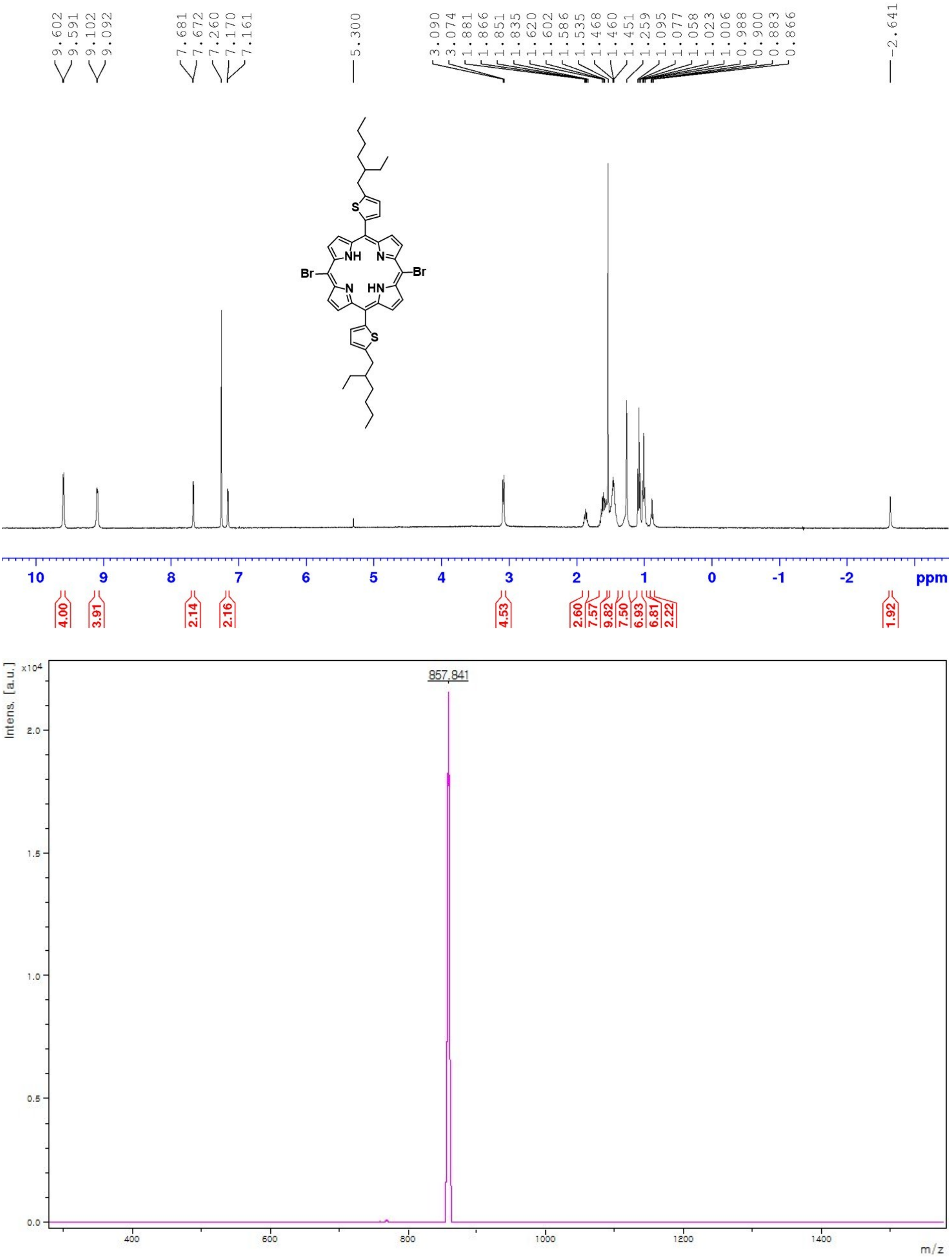

Figure S3. ^{1}H NMR (400 MHz, $CDCl_3$, 298 K) spectrum and MALDI-TOF-MS spectrum of compound **3**.

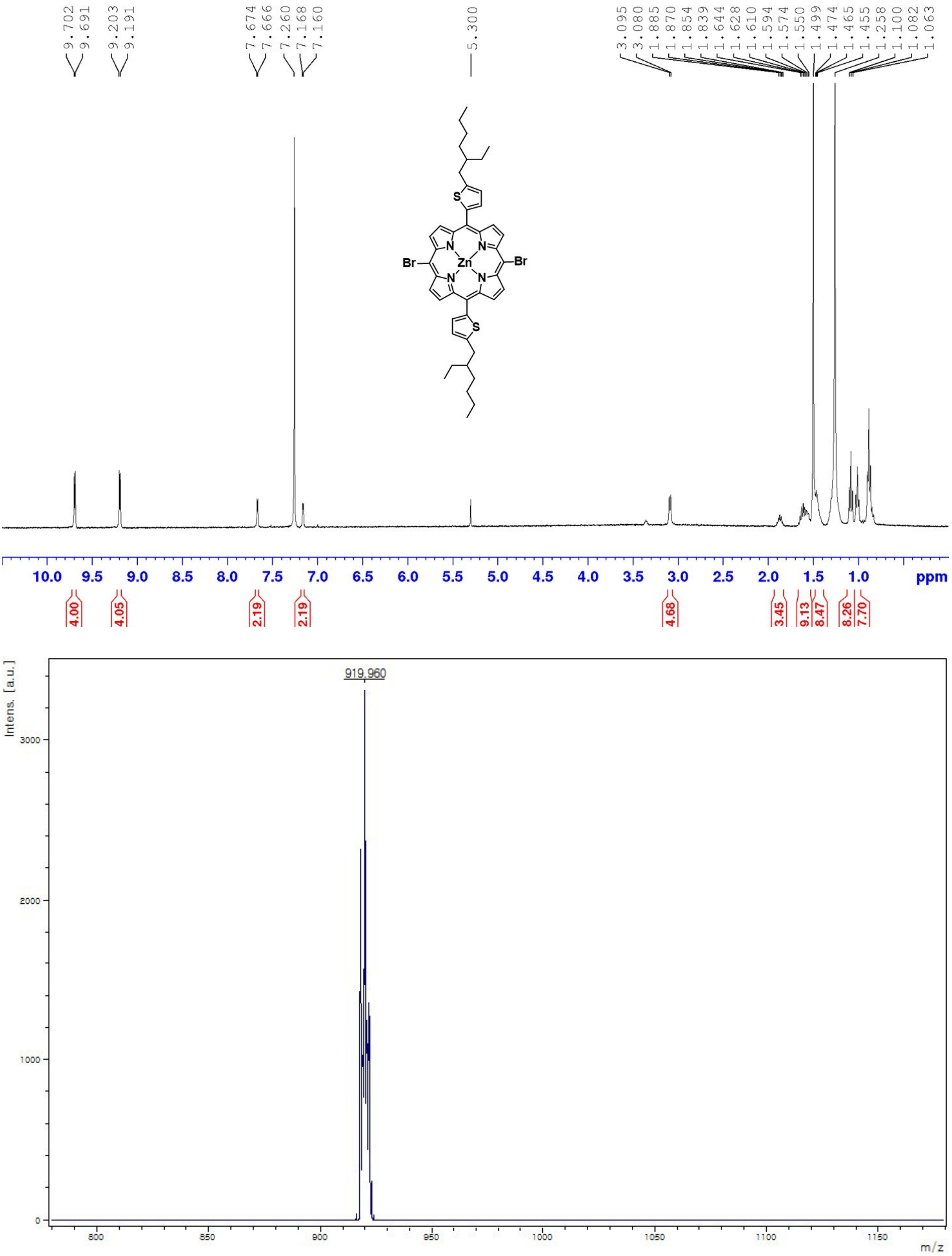

Figure S4. ^{1}H NMR (400 MHz, $CDCl_3$, 298 K) spectrum and MALDI-TOF-MS spectrum of compound **4**.

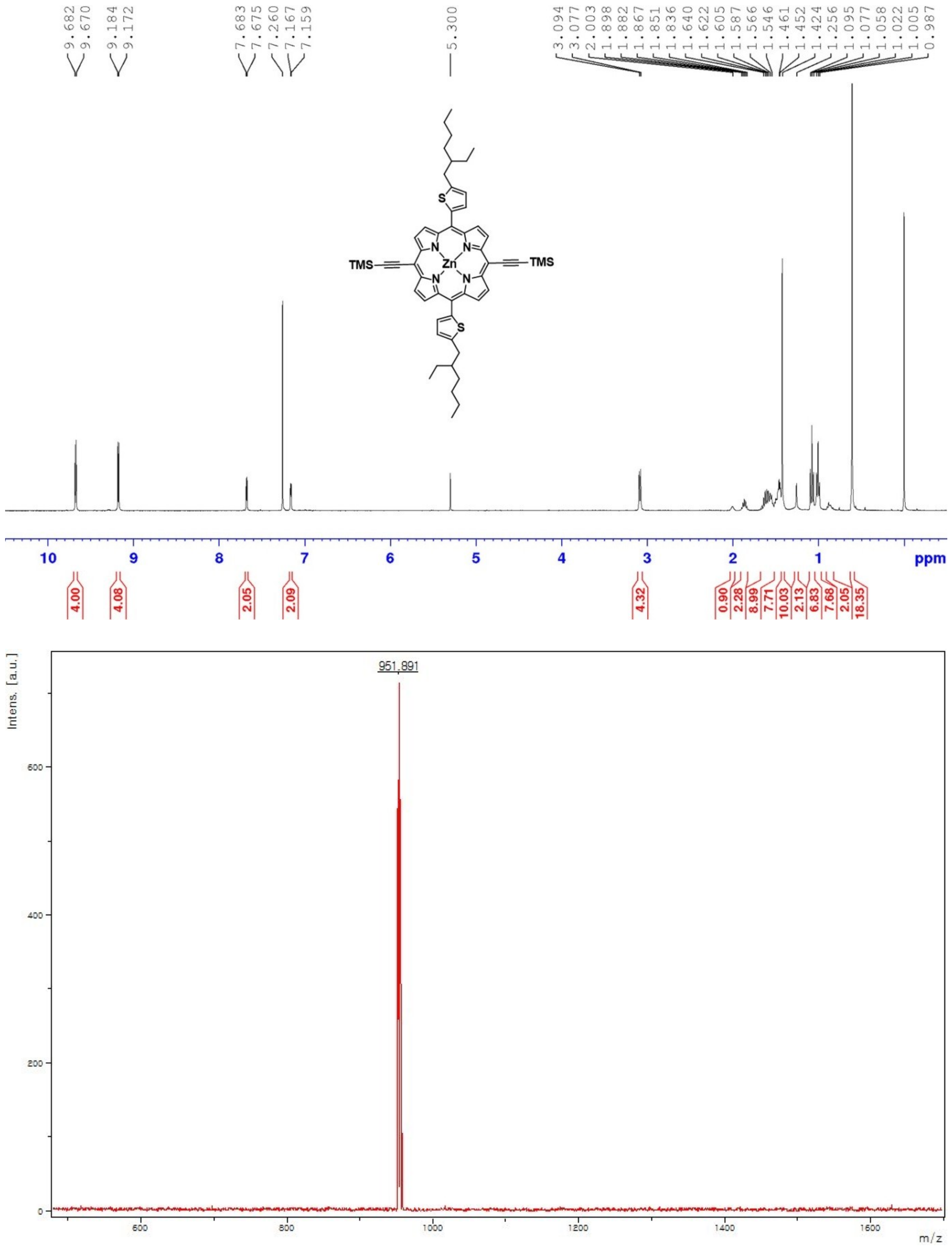

Figure S5. ^{1}H NMR (400 MHz, $CDCl_3$, 298 K) spectrum and MALDI-TOF-MS spectrum of compound **5**.

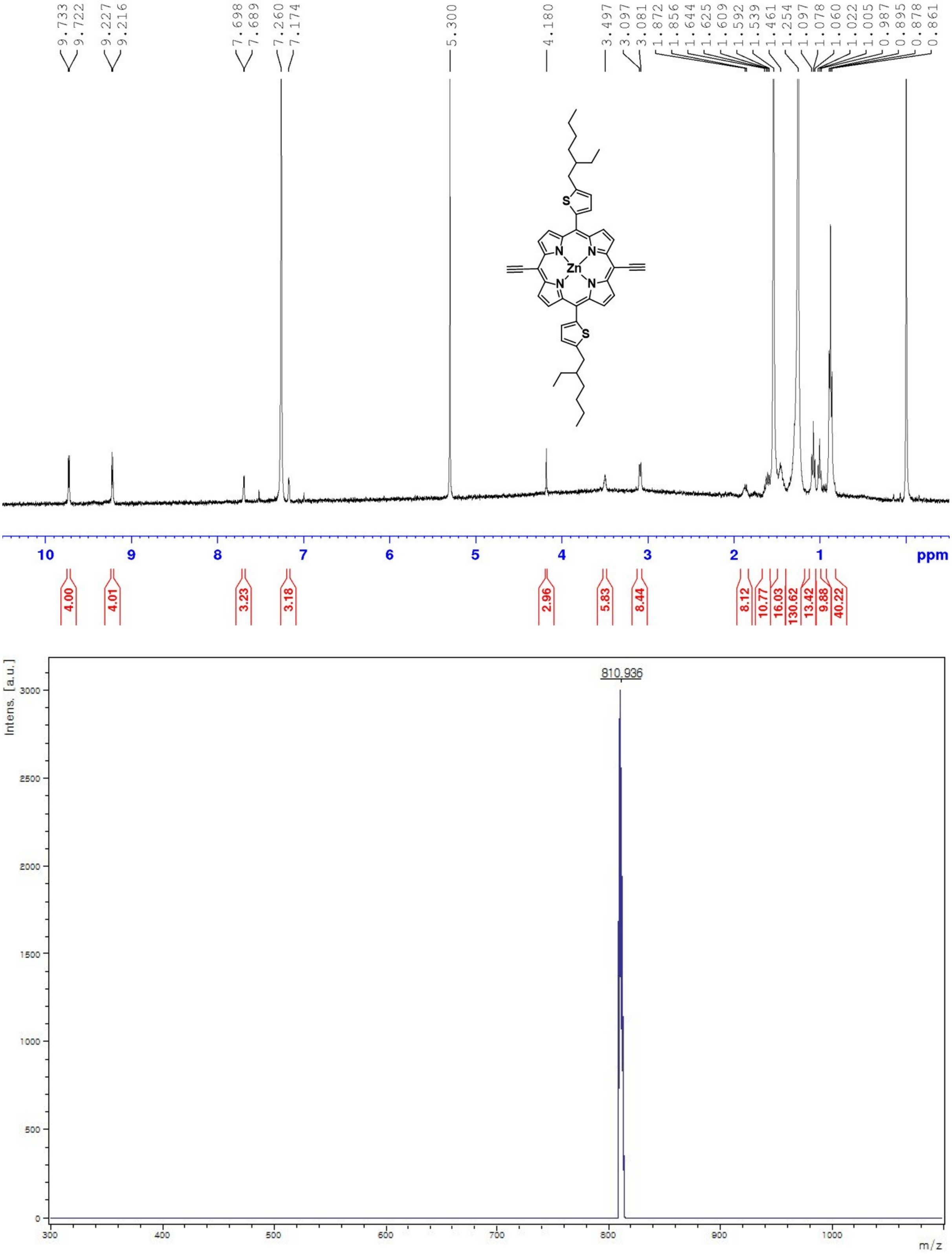

Figure S6. ^{1}H NMR (400 MHz, $CDCl_3$, 298 K) spectrum and MALDI-TOF-MS spectrum of compound **6**.

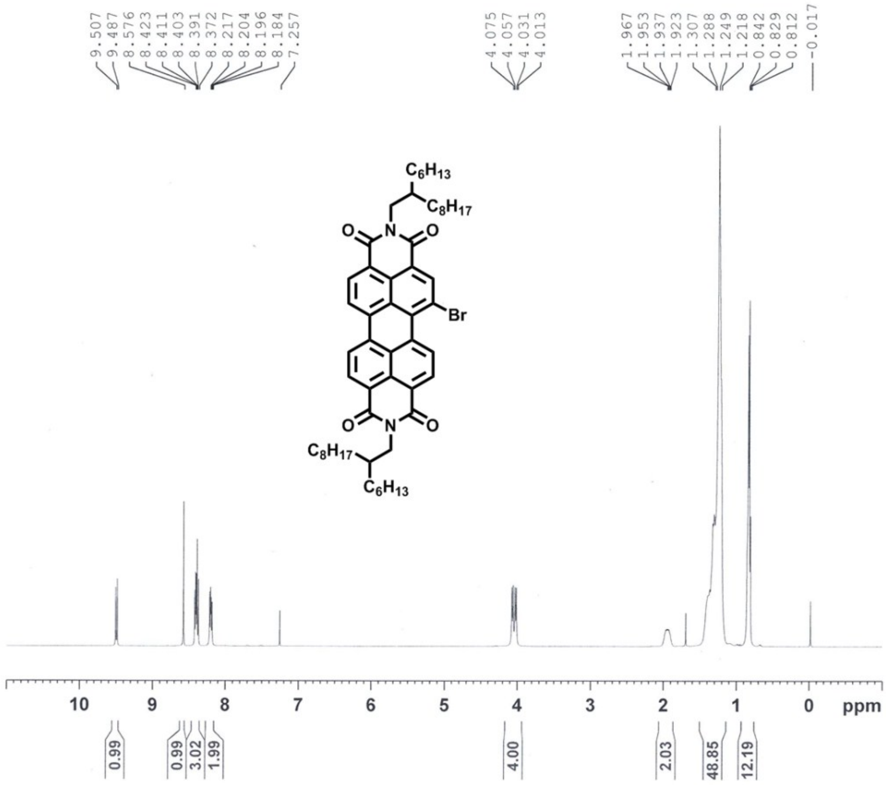

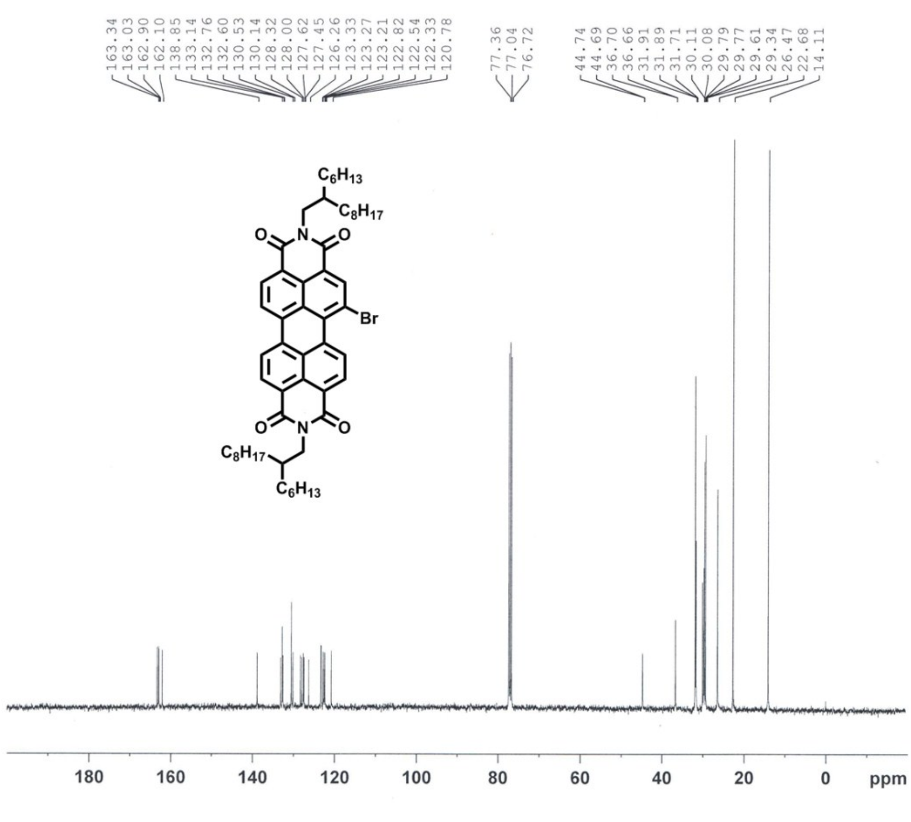

Figure S7. ^{1}H and ^{13}C NMR spectra of *1-bromo-N,N'-bis(2-ethylhexyl)-3,4,9,10-perylene tetracarboxylic diimide (PDI-Br)*.

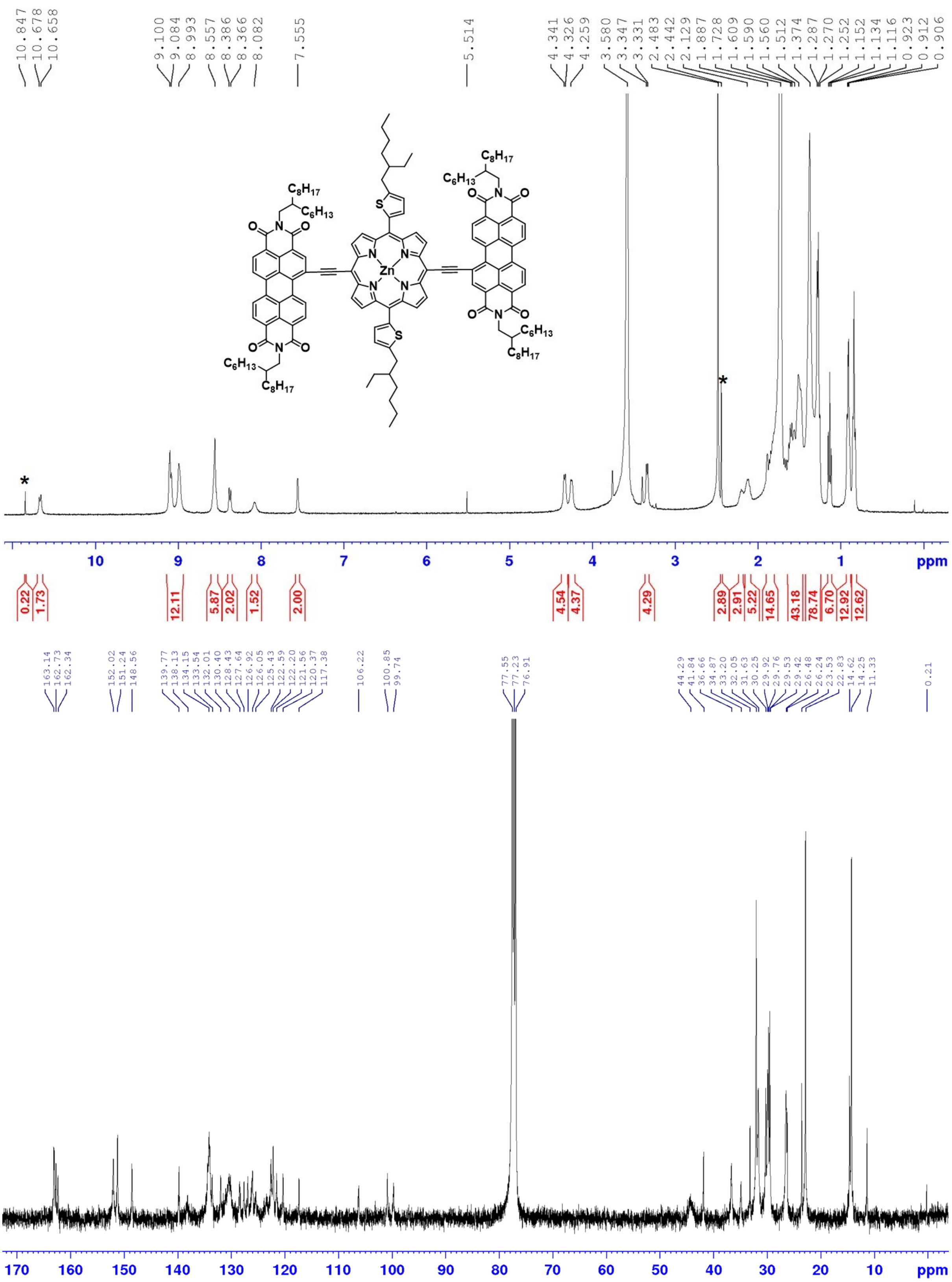

Figure S8. ^{1}H NMR (400 MHz, THF-d_8) and ^{13}C NMR (100 MHz, $CDCl_3$) spectra of PDI-P_{Zn}-PDI.

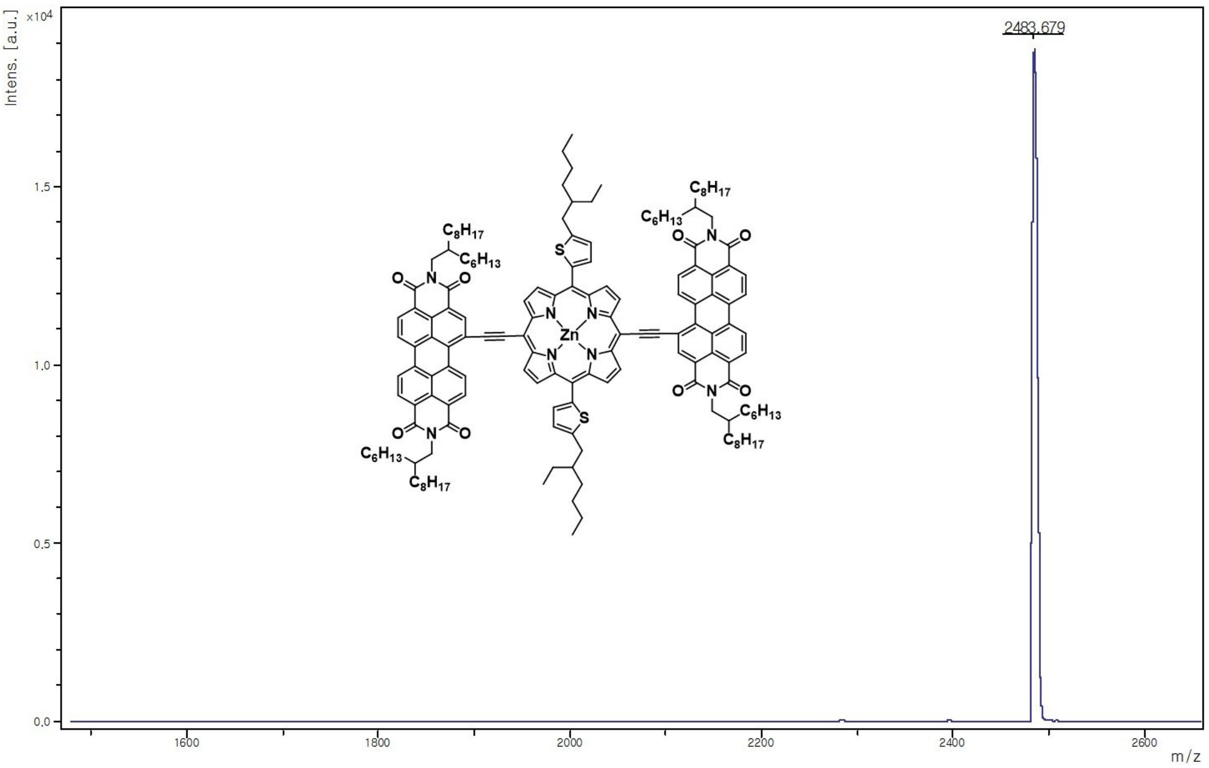

Figure S9. MALDI-TOF-MS spectrum of PDI-P_{Zn}-PDI.

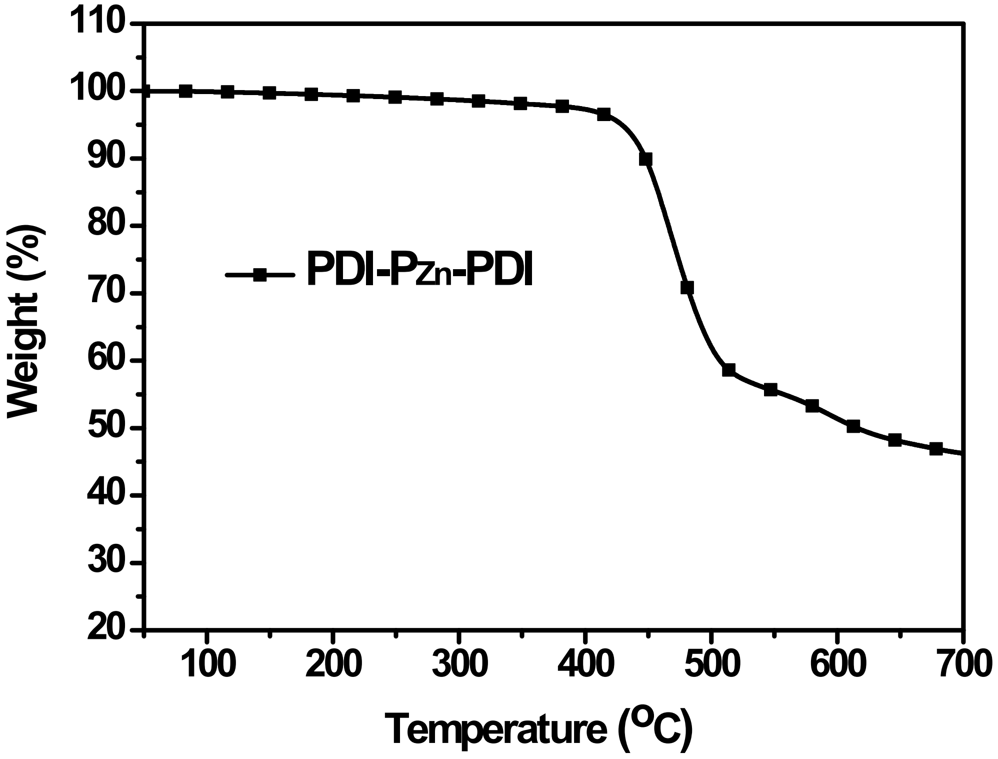

Figure S10. TGA thermogram of PDI-P_{Zn}-PDI

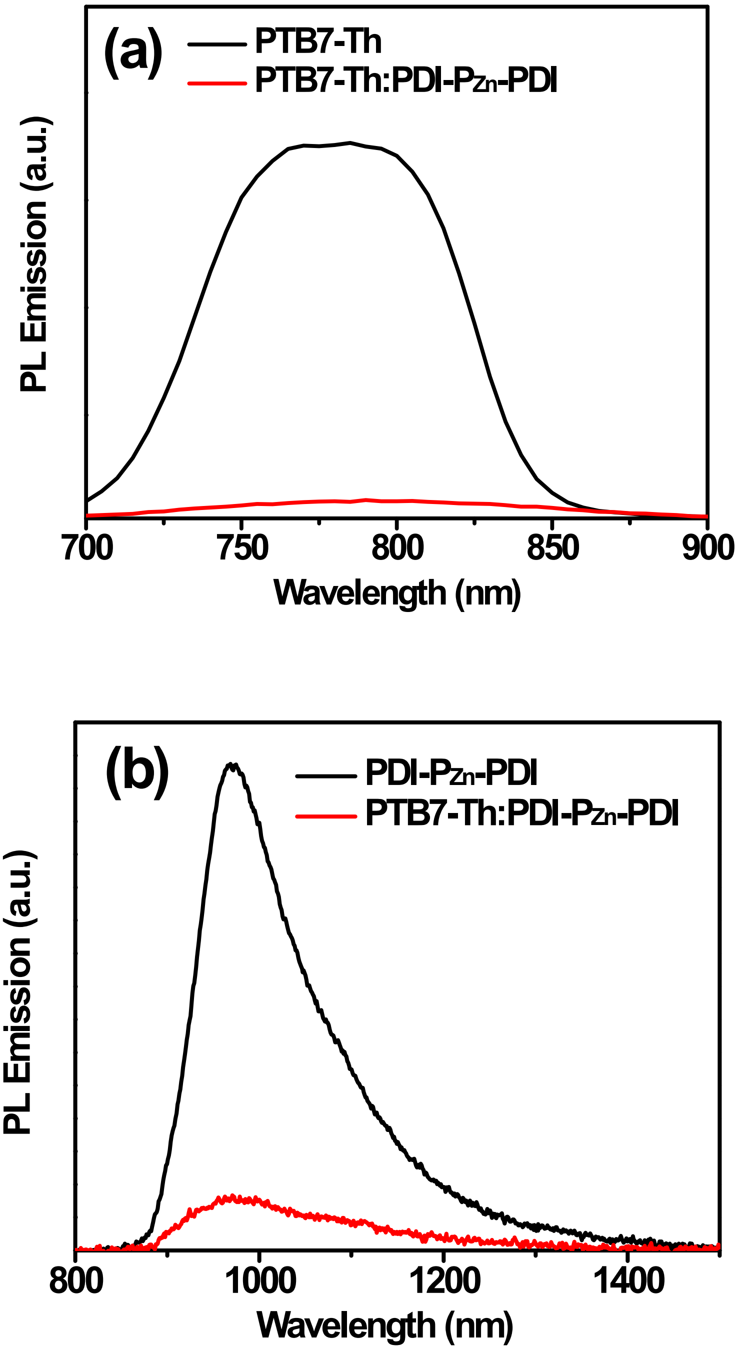

Figure S11. PL emission of (a) pristine PTB7-Th film and PTB7-Th:PDI-P_{Zn}-PDI blended film at an excitation of 650 nm (dominant excitation of PTB7-Th), and (b) pristine PDI-P_{Zn}-PDI film and PTB7-Th:PDI-P_{Zn}-PDI blended film at an excitation of 470 nm (dominant excitation of PDI-P_{Zn}-PDI acceptor)

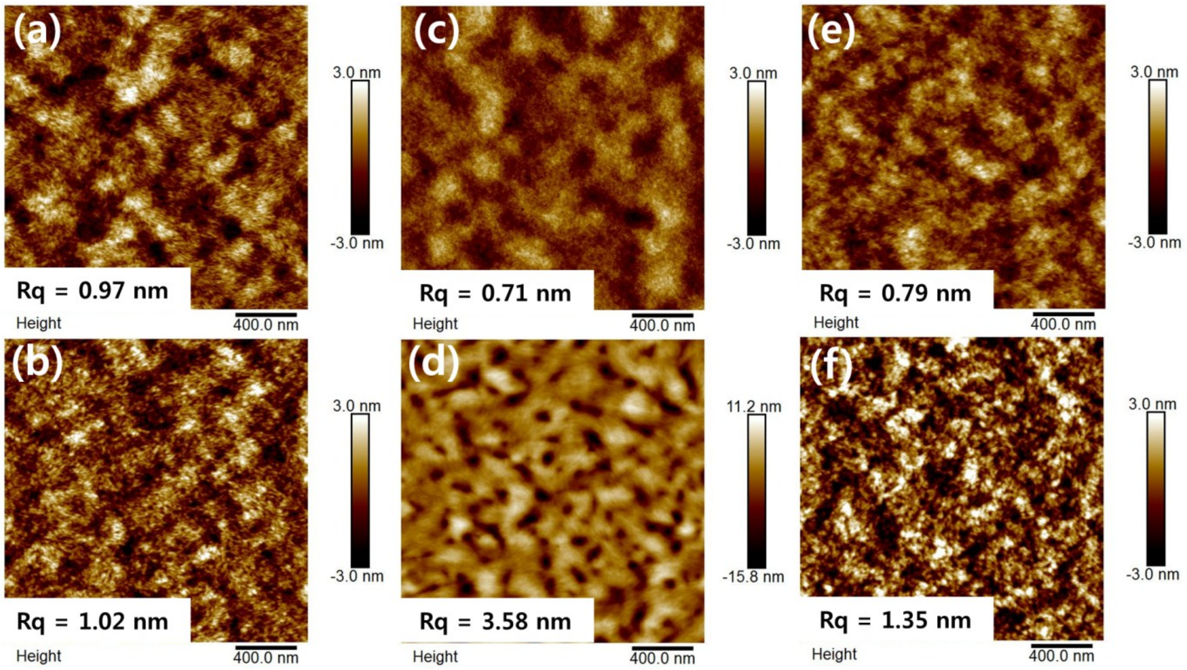

Figure S12. AFM images of a neat PTB7-Th film (a) w/o or (b) w/ pyridine, neat PDI-P_{Zn}-PDI film (c) w/o or (d) w/ pyridine, and PTB7-Th:PDI-P_{Zn}-PDI blended films in identically fabricated photovoltaic devices (e) w/o or (f) w/ pyridine.

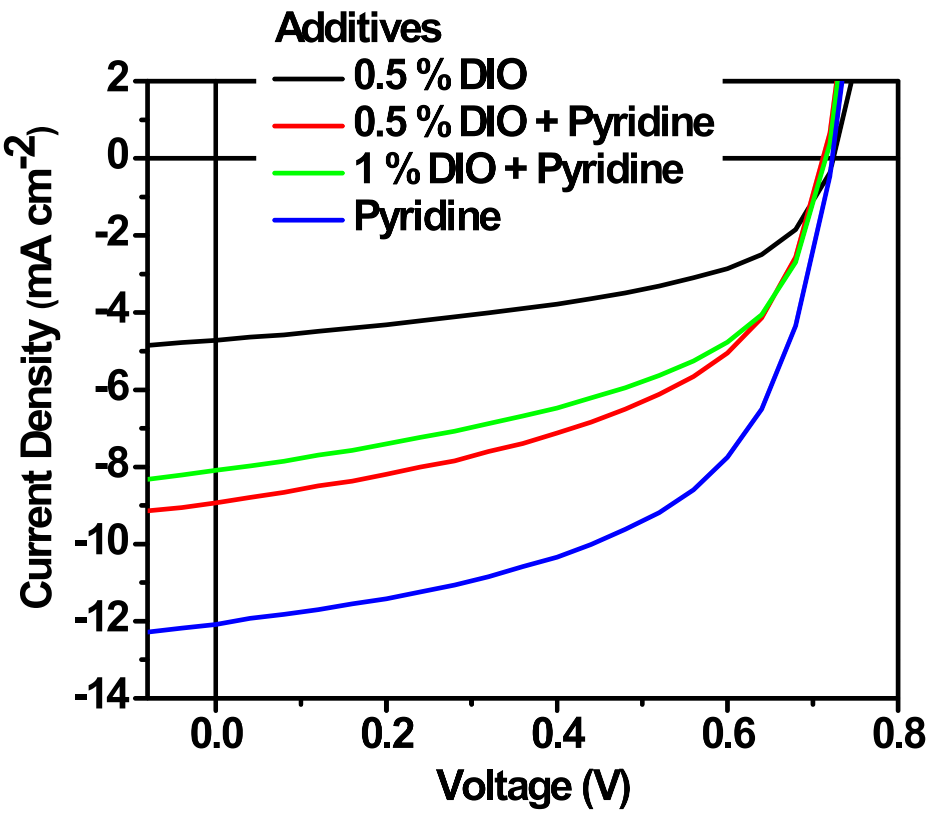

Figure S13. *J*-*V* characteristics of PTB7-Th:PDI-P_{Zn}-PDI photovoltaic devices according to various additives in the active layer.

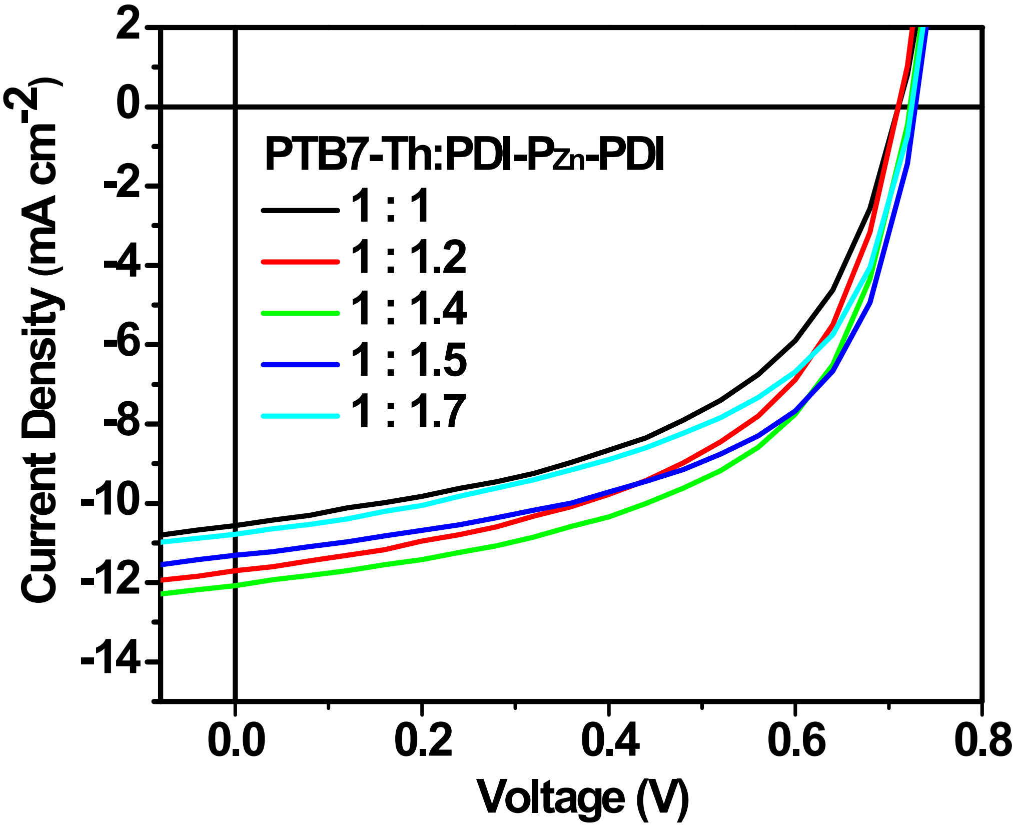

Figure S14. *J-V* characteristics of PTB7-Th:PDI-P_{Zn}-PDI photovoltaic devices at various donor/acceptor weight ratios.

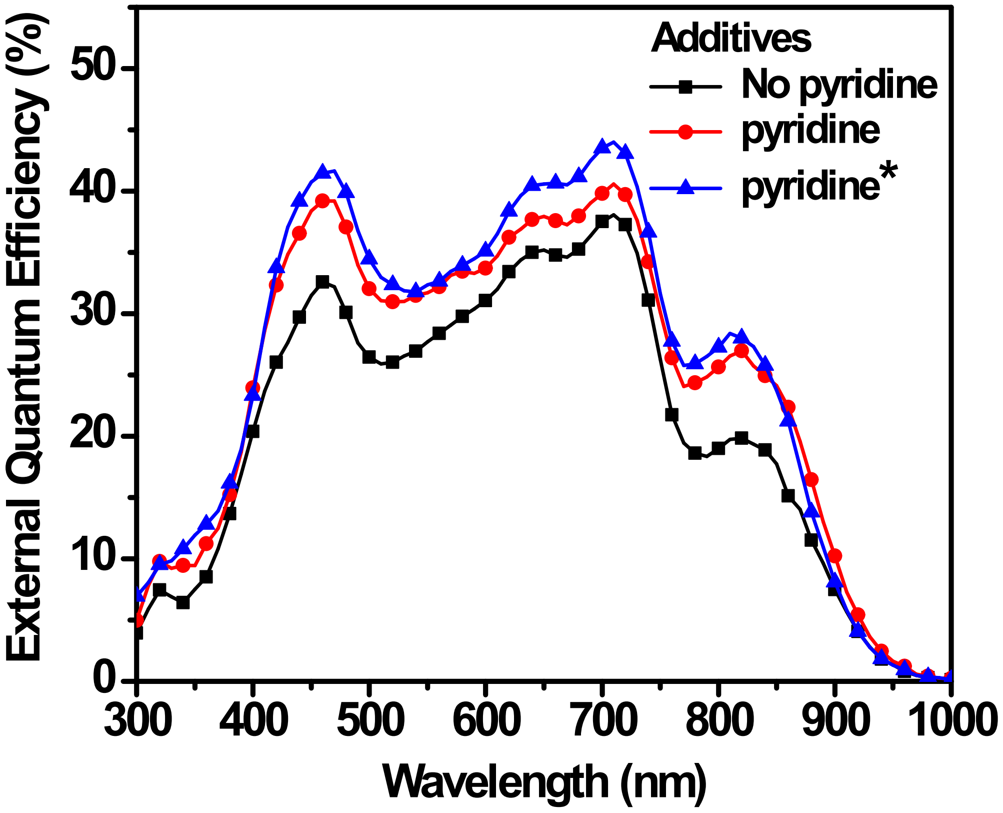

Figure S15. EQE of PTB7-Th:PDI-P_{Zn}-PDI photovoltaic devices corresponding to the J_{sc} values presented in Table 1.

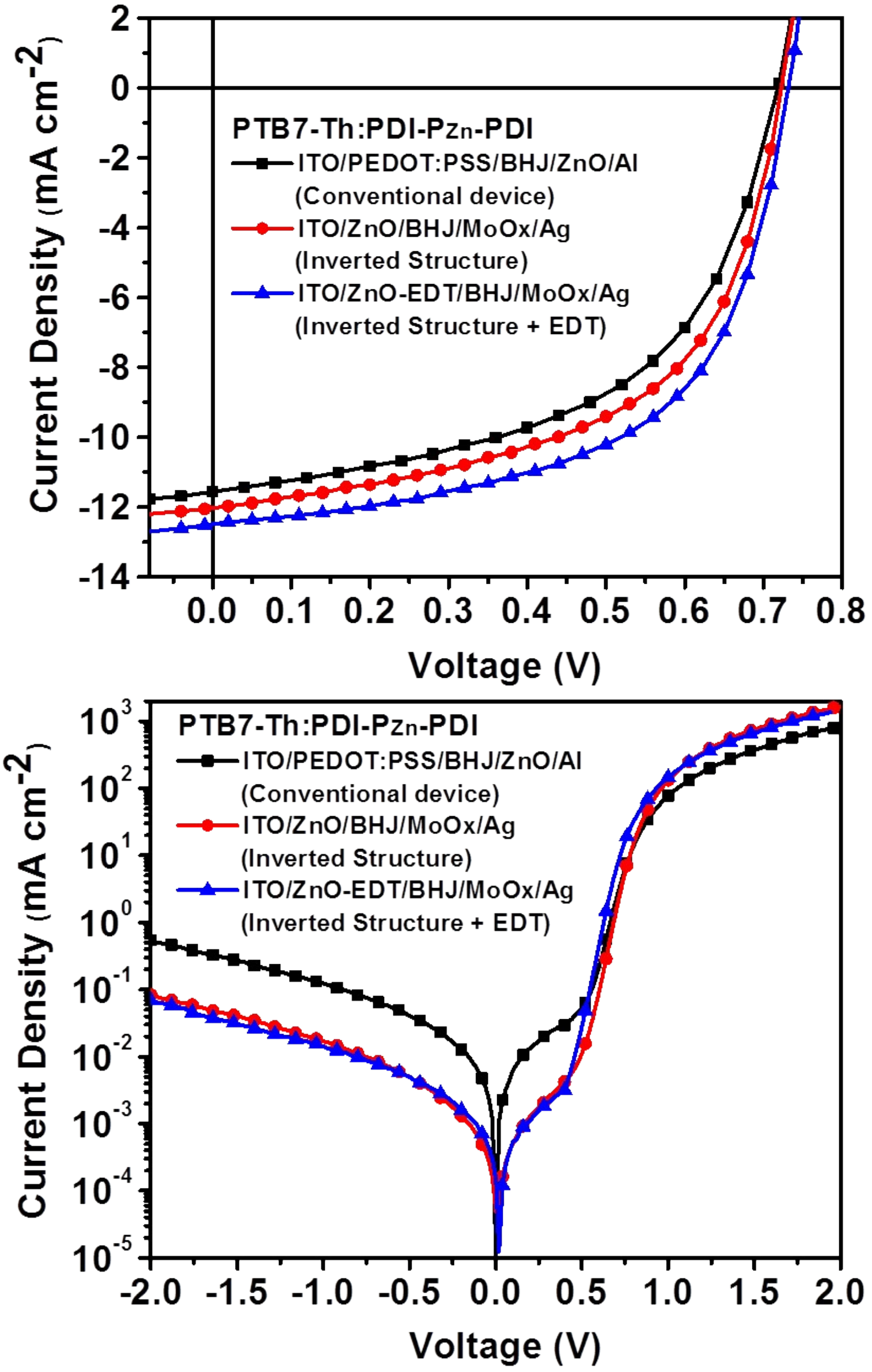

Figure S16. *J–V* characteristics of the inverted devices (ITO/ZnO/PTB7-Th:PDI-P_{Zn}-PDI/MoOx/Ag) and the conventional devices (ITO/PEDOT:PSS/PTB7-Th:PDI-P_{Zn}-PDI/ZnO/Al)

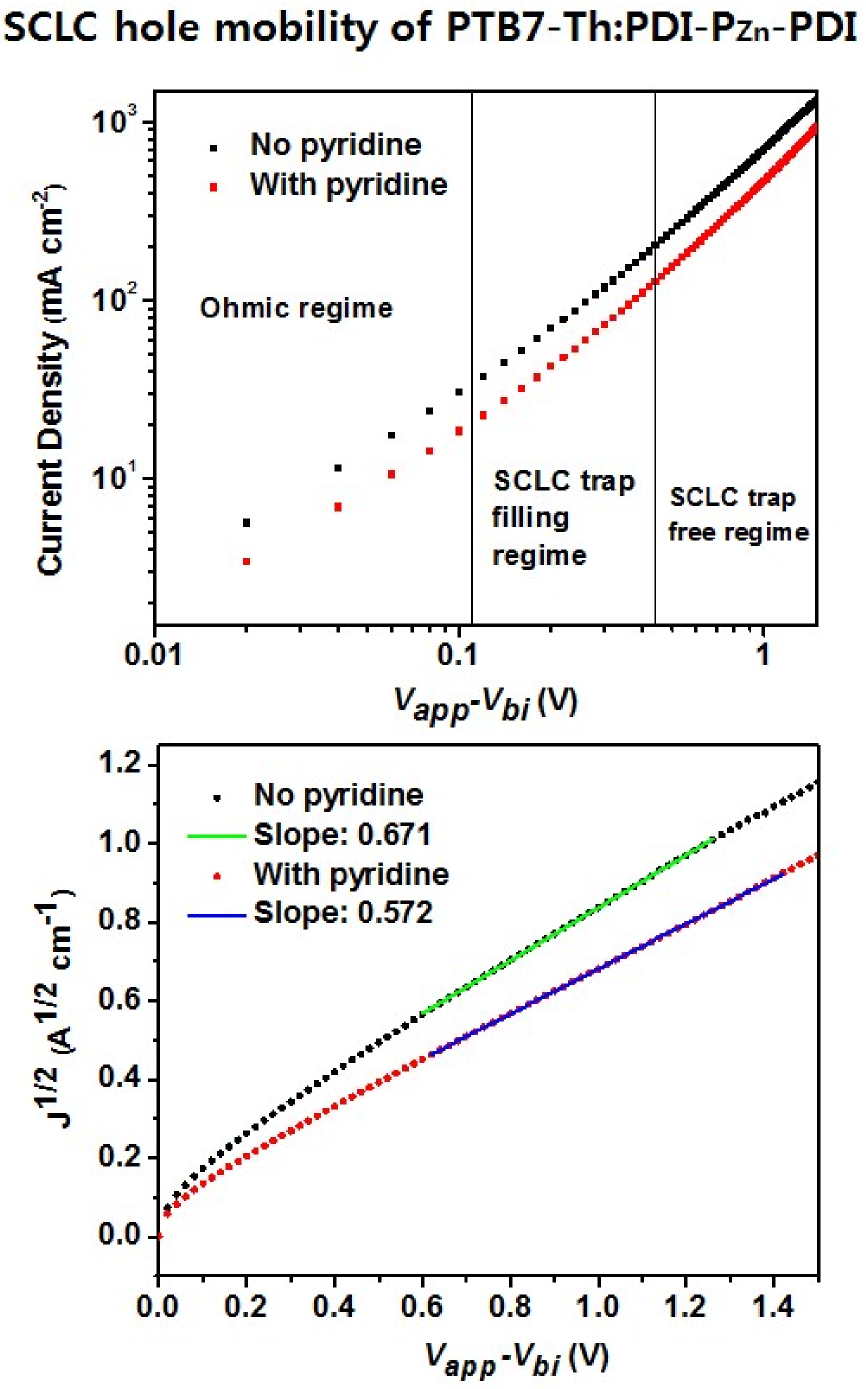

Figure S17. *J–V* characteristics of the hole-only devices with the PTB7-Th:PDI-P_{Zn}-PDI acceptor blended films w/ or w/o pyridine calculated by fitting the *J–V* curves in the SCLC regime.

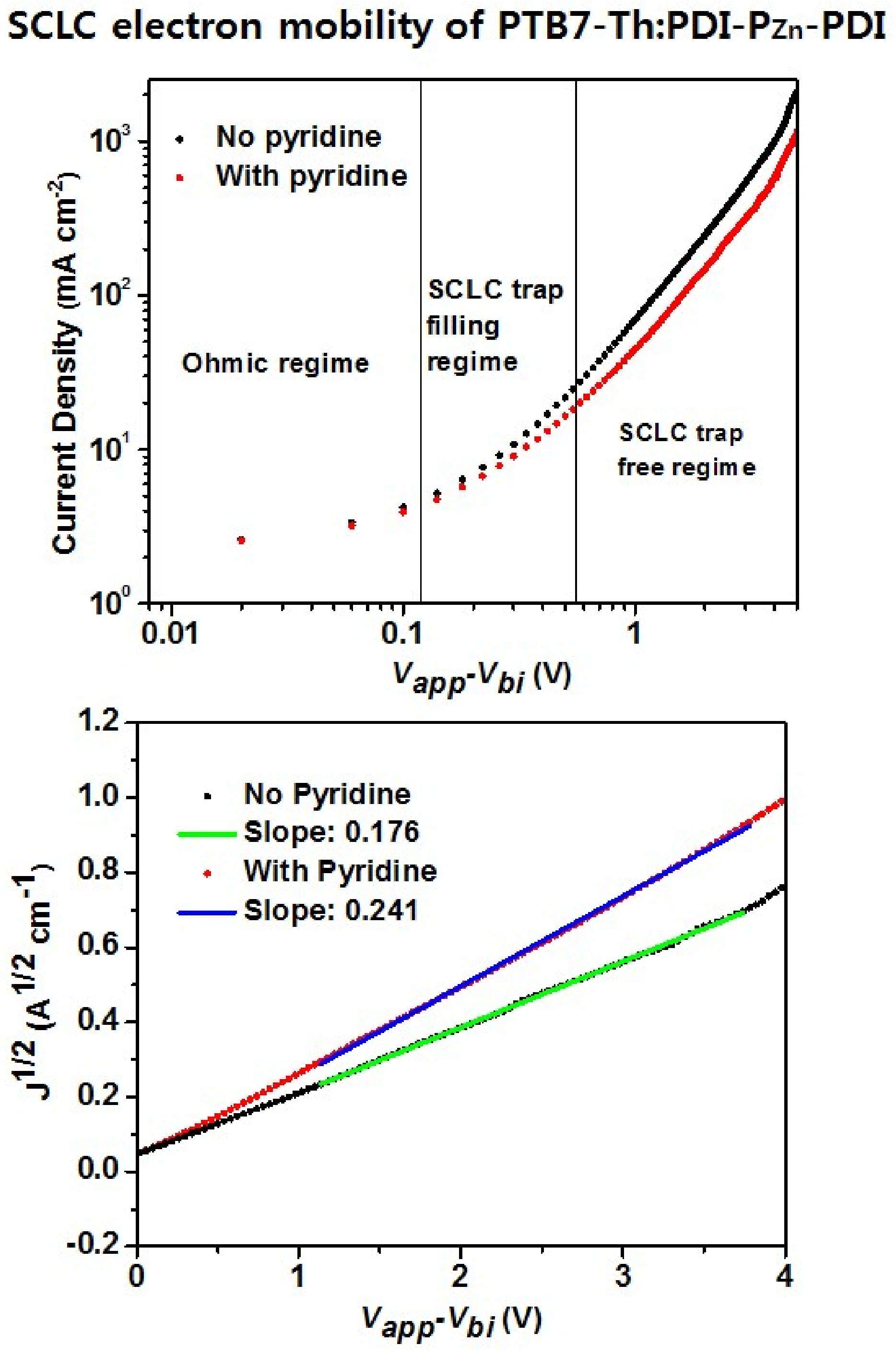

Figure S18. *J–V* characteristics of electron-only devices with the PTB7-Th:PDI-P_{Zn}-PDI acceptor blended films w/ or w/o pyridine, calculated by fitting the *J–V* curves in the SCLC regime.

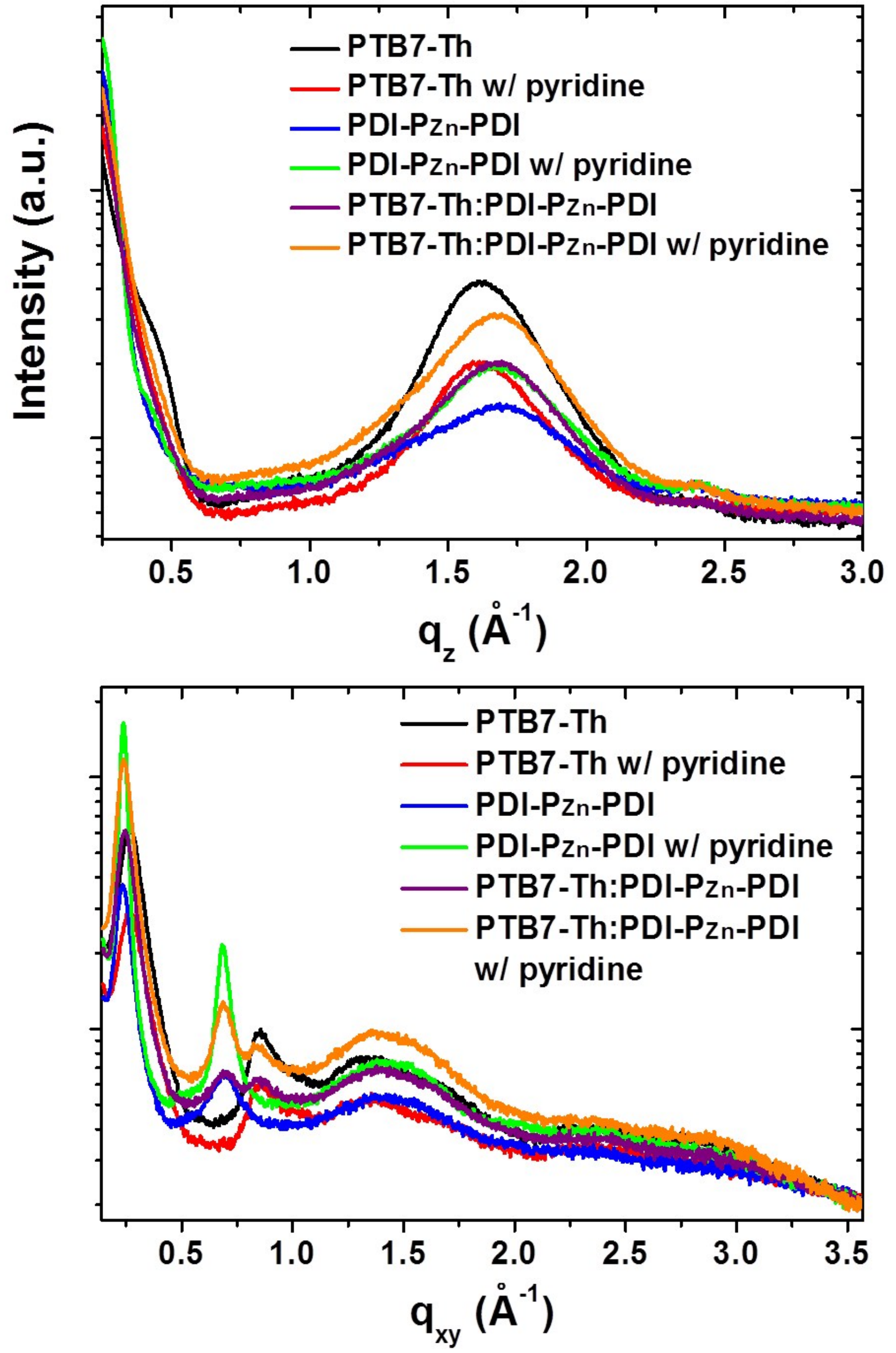

Figure S19. Out-of-plane and in-plane line cuts of neat PTB7-Th and PTB7-Th:PDI-P_{Zn}-PDI acceptor blended films.

Table S1. Summary of optical and electrochemical properties

	λ_{max} (nm)		E_g^{opt}	E_g^{CV}	E_{HOMO}	E_{LUMO}^{CV}	E_{LUMO}^{opt}
	solution[a]	film [b]	(eV)[c]	(eV)[d]	(eV)[e]	(eV)[f]	(eV)[g]
PTB7-Th	-	699	1.59	2.04	-5.23	-3.19	-3.64
$PC_{70}BM$	-	400, 504	1.91	2.41	-5.98	-3.57	-4.07
PDI-P_{Zn}-PDI	445, 780	467, 850	1.27	1.70	-5.50	-3.80	-4.23

[a] Dilute chloroform solution; [b] film on a quartz plate, formed by spin-coating a 1 wt% chloroform solution for 30 s at 1500 rpm; [c] bandgap calculated from the film-state absorption onset wavelength; [d] bandgap between E_{HOMO} and E_{LUMO}; [e] HOMO levels determined from E_{onset} of the first oxidation potential of ferrocene, −4.8 eV; [f] LUMO levels from E_{onset} of the first reduction potential; [g] LUMO levels calculated from HOMO levels and E_g^{opt}

Table S2. Summary of photovoltaic properties at various donor/acceptor weight ratios

donor:acceptor*	J_{SC} (mA cm^{-2})	V_{OC} (V)	Fill factor	PCE_{max} (ave) [%]
1:1	10.56	0.71	0.51	3.85
1:1.2	11.70	0.71	0.53	4.39
1:1.4	12.02	0.72	0.56	4.82
1:1.5	11.31	0.73	0.56	4.65
1:1.7	10.78	0.72	0.53	4.11
1:1	10.56	0.71	0.51	3.85

* The active layer was fabricated with 1% v/v added pyridine.

Table S3. Summary of photovoltaic properties with various additives

Additives	J_{SC} (mA cm^{-2})	V_{OC} (V)	Fill factor	PCE_{max} (ave) [%]
0.5% DIO	4.72	0.72	0.51	1.74
0.5% DIO + pyridine	8.93	0.71	0.50	3.18
1% DIO + pyridine	8.09	0.72	0.51	2.94
pyridine	12.02	0.72	0.56	4.82

* The active layer was composed of PTB7-Th:PDI-P_{Zn}-PDI at a weight ratio of 1:1.4.

Table S4. Summary of photovoltaic properties of conventional and inverted devices

Structure	Structure	Jsc (mA·cm^{-2})	Voc (V)	FF	PCE (%)
ITO/PEDOT:PSS/BHJ /ZnO/Al	Conventional	11.55	0.72	0.53	4.42
ITO/ZnO/BHJ/MoOx/Ag	Inverted	12.02	0.72	0.56	4.82
ITO/ZnO with EDT/BHJ /MoOx/Ag[a]	Inverted	12.49	0.73	0.58	5.28

* The active layer was composed of PTB7-Th:PDI-P_{Zn}-PDI at a weight ratio of 1:1.4.
[a] ZnO ETL was treated by EDT

References

S1. Park, H.-Y.; Ryu, I.; Kim, J.; Jeong, S.; Yim, S.; Jang, S.-Y., PbS Quantum Dot Solar Cells Integrated with Sol–Gel-Derived ZnO as an n-Type Charge-Selective Layer. *J. Phys. Chem. C* **2014,** 118, (31), 17374-17382.

S2. Sekine, N.; Chou, C.-H.; Kwan, W. L.; Yang, Y., ZnO nano-ridge structure and its application in inverted polymer solar cell. *Org. Electronics* **2009,** 10, (8), 1473-1477.
